# Ultrafiltered Mulberry (*Morus alba* L.) Leaf Albumin-Type Protein Attenuates High-Fat Diet-Induced Obesity in Mice by Remodeling Gut Microbiota and Metabolic Homeostasis

**DOI:** 10.3390/foods15162774

**Published:** 2026-08-07

**Authors:** Leyi Yu, Kaiwen Luo, Dongjun He, Guoxing Yu, Yu Yang, Hong Yao, Chongzhen Sun, Xiyang Wu

**Affiliations:** 1 School of Life Sciences, Zhejiang Chinese Medical University, Hangzhou 310053, China; 20241156@zcmu.edu.cn; 2 Department of Food Science and Engineering, Jinan University, Guangzhou 510632, China; 15728054368@163.com (K.L.); 13202457231@163.com (D.H.); cusiyu@163.com (G.Y.); 13427519502@163.com (Y.Y.); whyhsss@outlook.com (H.Y.); 3 School of Public Health, Guangdong Pharmaceutical University, Jianghai Avenue 283, Haizhu District, Guangzhou 510006, China

**Keywords:** mulberry leaf albumin-type protein, obesity, gut microbiota, metabolomics

## Abstract

Obesity is a chronic metabolic disorder closely associated with dyslipidemia, insulin resistance, low-grade inflammation, and gut microbiota dysbiosis. Mulberry leaves are rich in bioactive proteins, but whether mulberry leaf albumin-type protein can improve diet-induced obesity remains unclear. In this study, ultrafiltered mulberry leaf albumin-type protein (UMP) was prepared and its anti-obesity effects were evaluated in high-fat diet (HFD)-fed C57BL/6J mice. UMP contained 87.12 ± 0.52 g/100 g protein, 2.52 ± 0.00 g/100 g polyphenols, and 8.21 ± 1.49 g/100 g polysaccharides, with two major albumin-type protein bands of approximately 14 and 52 kDa. Structural analysis showed that UMP was mainly composed of *β*-turns and *α*-helices. In HFD-fed mice, daily administration of UMP for 16 weeks reduced body weight gain by 3.85 g and 5.63 g in the low- and high-dose groups, respectively, without affecting food intake. Biochemical assays, glucose and insulin tolerance tests, and histological analysis showed that UMP improved insulin responsiveness, alleviated serum dyslipidemia, reduced hepatic lipid accumulation, and decreased circulating alanine aminotransferase, aspartate aminotransferase, and lipopolysaccharide levels. Histological analysis and nuclear magnetic resonance-based short-chain fatty acid quantification further showed that UMP protected colonic morphology and increased colonic short-chain fatty acid levels. Gut microbiota analysis showed that UMP restored microbial diversity, reduced the Firmicutes/Bacteroidota ratio, and enriched potentially beneficial genera, including *Ileibacterium* and *norank*_*f*_*Muribaculaceae*. Fecal biochemical assays suggested that UMP promoted fecal free fatty acid excretion and partially improved bile acid-related metabolic alterations. Untargeted serum metabolomics revealed that UMP reshaped metabolic pathways related to lipid turnover, bile acid signaling, glucose utilization, and glucuronidation. Correlation analysis linked UMP-enriched bacterial taxa with key metabolites involved in fatty acid and energy metabolism. Together, these findings indicate that UMP attenuates HFD-induced obesity through coordinated regulation of gut microbiota, intestinal metabolites, and systemic metabolic homeostasis. UMP may therefore represent a promising functional dietary protein for the prevention of obesity-related metabolic disorders.

## 1. Introduction

The prevalence of obesity has risen dramatically over the past few decades and is now considered a global epidemic [1]. Obesity is a chronic, multifactorial disease characterized by excessive adipose tissue accumulation that adversely affects health, increasing the risk of type 2 diabetes, cardiovascular disease, certain cancers, and impairing bone health and reproductive function [2,3]. As a major global health challenge, the condition offers only limited therapeutic options, which are primarily restricted to dietary modification, physical activity, pharmacotherapy, and bariatric surgery [4]. Lifestyle modification generally results in modest weight reduction, which is frequently followed by gradual weight regain driven by metabolic adaptation [5]. Pharmacotherapy, including agents such as orlistat, liraglutide, semaglutide, and tirzepatide, represents an important adjunct to obesity management, demonstrating efficacy but carrying the potential for adverse effects [6,7].

Recent advances have revealed that the gut microbiota functions as a pivotal environmental factor linking diet, host metabolism, and metabolic health [8]. The gut microbial community not only regulates nutrient absorption and energy harvest but also generates a wide range of bioactive metabolites that enter the circulation and influence systemic metabolic homeostasis [9]. Consequently, dysbiosis-the imbalance in gut microbial composition and function-has been implicated as a major contributor to the onset and progression of obesity and its metabolic complications. Notably, correlations between specific microbes and serum metabolites, such as *Faecalibacterium* with butyrate or *Bacteroides* with bile acid derivatives, have been linked to insulin resistance and adiposity phenotypes [8]. Among bioactive dietary components, dietary proteins have gained attention for their capacity to modulate gut microbiota and improve host metabolic health [10]. Evidence from a murine model has shown that dietary proteins from diverse sources, including soy, egg white, and pea, can markedly reshape gut microbial communities and alter microbial amino acid and glycosylations metabolic pathways, thereby exerting profound effects on microbiota-driven diseases [11]. In line with these findings, supplementation with plant-based protein such as pea protein extracts increased the abundance of beneficial commensal genera such as *Akkermansia* and *Parabacteroides*. Moreover, Pea albumin suppressed lipid accumulation by inhibiting adipogenesis and enhancing fatty-acid oxidation [12].

Mulberry (*Morus alba* L.) is a traditional medicinal and edible plant in China and a potential source of various bioactive compounds [13]. Apart from serving as the primary feed source for silkworms, mulberry leaves also function as a valuable herbal medicine and healthy food material [14]. In 1993, the Ministry of Health of the People’s Republic of China officially listed mulberry leaves within the catalogue of substances with homology of medicine and food [15]. As a typical medicinal-edible plant, mulberry leaves have gained widespread acknowledgment owing to their prominent application value across the food and pharmaceutical industries [16]. The leaves of mulberry exhibit diverse pharmacological activities, including antihyperglycemic, antioxidant, lipid-lowering, anticancer, and anti-inflammatory effects [17]. Notably, the leaves contain a relatively high protein content, ranging from approximately 17% to 25%, which contributes substantially to their nutritional and functional value [18,19]. Additionally, mulberry leaf protein (MLP) possesses a well-balanced amino acid profile comprising all eight essential amino acids, with an essential amino acid index (EAAI) surpassing that of conventional feed proteins [20]. Our previous studies have demonstrated that mulberry leaf protein (MLP) possesses potent antioxidant and cytoprotective activities, effectively scavenging free radicals and mitigating oxidative stress [21,22,23]. However, as a protein resource with considerable development potential, the anti-obesity properties of MLP remain largely unexplored.

This study first characterized the chemical composition and structure of UMP, then evaluated its effects on HFD-induced obese mice. Our integrated approach involved analyzing body weight, glucose metabolism, serum biochemistry, and histopathology; characterizing gut microbiota via 16S rRNA sequencing; and exploring metabolic reprogramming via untargeted serum metabolomics. Together, these findings provide scientific evidence to support the high-value development and utilization of MLP.

## 2. Material and Methods

### 2.1. Chemicals and Reagents

n-Heptane and absolute ethanol were purchased from Guangzhou Guanghua Technology Co., Ltd. (Guangzhou, China). D-glucose was supplied by Shanghai YuanYe Biotechnology Co., Ltd. (Shanghai, China), and recombinant human insulin was obtained from Procell Life Science & Technology Co., Ltd. (Wuhan, China). The mouse lipopolysaccharide (LPS) ELISA kit was purchased from Cusabio Biotech Co., Ltd. (Wuhan, China). Total bile acid (TBA), total cholesterol (TC), and triglyceride (TG) assay kits were provided by Nanjing Jiancheng Bioengineering Institute (Nanjing, China). The free fatty acid (FFA) assay kit was purchased from Abbkine Scientific Co., Ltd. (Wuhan, China). FastPure Stool DNA Isolation Kit was provided from Tiangen Biotech Co., Ltd. (Beijing, China). The bicinchoninic acid (BCA) protein assay kit was obtained from Beyotime Biotechnology (Shanghai, China).

### 2.2. Preparation of UMP

Mulberry leaves collected from Huadu District, Guangzhou, China, were oven-dried and milled into a fine powder passing through a 60-mesh sieve. Mulberry leaf protein was extracted according to the procedure described by He et al. [19] with minor modifications. In brief, the powder was suspended in deionized water (1:15, *w*/*v*), homogenized, and subjected to ultrasonication for 15 min (15 kHz, 350 W). The suspension was subsequently stirred at room temperature for 2 h and centrifuged at 8000× *g* for 15 min at 4 °C. The supernatant was filtered through 200-mesh gauze to obtain the protein-rich extract. The pH of the extract was then adjusted to 3.5 with 1 M HCl to induce protein precipitation. Following a 30 min standing period, the precipitate was obtained by centrifugation at 12,000× *g* for 20 min at 4 °C, subsequently redissolved in deionized water, and the pH was adjusted to neutrality using 1 M NaOH. The solution was desalted using a dialysis membrane (MWCO: 4.5 kDa) and subsequently lyophilized to yield crude mulberry leaf protein, which was stored at −20 °C until further analysis.

Crude mulberry leaf protein (MP) was further purified using an ultrafiltration membrane technique. A crude protein solution (1 mg/mL) was prepared in deionized water, and insoluble impurities were removed by vacuum filtration. The resulting supernatant was subjected to ultrafiltration with Amicon^®^ Ultra-15 (Merck KGaA, Darmstadt, Germany) centrifugal filter units (10 kDa molecular weight cut-off, MWCO) by centrifugation (1500× *g*, 20 min) at 4 °C. The retained fraction was collected, freeze-dried, weighed, and designated as ultrafiltered mulberry leaf albumin-type protein (UMP). The recovery yield of the final UMP fraction after ultrafiltration was calculated as the dry weight of lyophilized UMP divided by the dry weight of crude mulberry leaf protein used for ultrafiltration.

### 2.3. Analysis of Composition and Characterization

The proximate composition of UMP was characterized to assess its nutritional and functional attributes. Protein concentration was measured using bicinchoninic acid (BCA) assay [24], total sugar content was determined using the phenol-sulfuric acid method [25], and total phenolic content was evaluated with the Folin–Ciocalteu method [26].

The molecular weight distribution of the MP was analyzed by sodium dodecyl sulfate-polyacrylamide gel electrophoresis (SDS–PAGE) [27]. Briefly, protein samples were mixed with loading buffer containing 2% SDS and 5% *β*-mercaptoethanol, followed by denaturation at 100 °C for 10 min using a metal block heater to ensure complete unfolding of the proteins. Approximately 15 µL of each sample (containing 300 µg protein) was loaded onto a 12% resolving gel with a 5% stacking gel. Electrophoresis was performed at 80 V for stacking and 120 V for separation until the dye front reached the bottom. Gels were stained with Coomassie Brilliant Blue R-250 and destained in a methanol-acetic acid solution. Molecular weight markers (10–250 kDa) were used as standards.

The structural characteristics of the UMP were analyzed by Fourier transform infrared (FTIR) spectroscopy using an FTIR spectrometer (Nicolet iS50, Thermo Scientific, Waltham, MA, USA) [28]. The freeze-dried protein powder was mixed with spectroscopic-grade potassium bromide (KBr) at a ratio of 1:150 (*w*/*w*) and pressed into a translucent pellet under 10 MPa. Spectra were recorded in the range of 4000–400 cm^−1^ with a resolution of 4 cm^−1^ and 32 scans per sample. Background spectra were collected and automatically subtracted. The amide I region (1600–1700 cm^−1^) was deconvoluted to analyze secondary structure components (*α*-helix, *β*-sheet, *β*-turn, and random coil) by Gaussian fitting.

### 2.4. Design of Animal Experiment

Male C57BL/6J mice (6 weeks of age, body weight 20 ± 2 g) were obtained from Guangdong Yaokang Biotechnology Co., Ltd. (Guangzhou, China). All experimental procedures were performed in accordance with the Guidelines for the Care and Use of Laboratory Animals of Jinan University and approved by the Institutional Animal Care and Use Committee (IACUC Approval No. IACUC-20240521-05).

Mice were maintained under standardized conditions (temperature: 21 ± 1 °C; relative humidity: 50 ± 5%) with a 12-h light/dark cycle and had ad libitum access to standard chow and water. Following a 7-day acclimatization period, mice were randomly assigned to four groups (*n* = 7 per group): normal diet group (ND), high-fat diet group (HFD), low-dose UMP intervention group (LUMP), and high-dose UMP intervention group (HUMP). The ND group was fed with a standard chow diet (10% of total energy from fat), whereas the HFD, LUMP, and HUMP groups received a high-fat diet (60% of total energy from fat). In addition, mice in the LUMP and HUMP groups were administered UMP by daily oral gavage at doses of 100 mg/kg and 300 mg/kg body weight, respectively, while the ND and HFD groups received the same volume of sterile water. The doses 100 mg kg^−1^ day^−1^ and 300 mg kg^−1^ day^−1^ in the UMP groups were separately equivalent to 8.1 and 24.3 mg kg^−1^ daily for humans (60 kg) calculated based on the body surface area [29]. Body weight and food intake were measured weekly throughout the 16-week experimental period.

### 2.5. Glucose and Insulin Tolerance Tests (OGTT and ITT)

To evaluate glucose metabolism and insulin sensitivity, an oral glucose tolerance test (OGTT) was conducted in week 15 after a 16 h fast, during which mice received glucose (2 g/kg, gavage). Tail vein blood glucose levels were measured at 0, 30, 60, 90, and 120 min post-administration, and the area under the curve (AUC) was calculated. In week 16, an insulin tolerance test (ITT) was performed following a 6 h fast, with mice intraperitoneally injected with insulin (0.8 U/kg). Blood glucose was measured at the same time points, and AUC values were determined.

### 2.6. Sample Collection and Tissue Processing

At week 16, fresh fecal samples were collected and stored at −80 °C. After a 12 h fast, blood was collected via the orbital sinus into heparinized tubes, and mice were euthanized by cervical dislocation. Blood samples were centrifuged at 3500 rpm for 10 min at 4 °C, and serum was aliquoted and stored at −80 °C. Liver, colon, and other tissues were excised, weighed, and either fixed in 4% paraformaldehyde for histological analysis or snap-frozen in liquid nitrogen and stored at −80 °C. Colonic contents were also collected, snap-frozen, and stored at −80 °C.

### 2.7. Histopathological Evaluation

Colon and liver tissues were fixed in 4% paraformaldehyde for 24 h, paraffin-embedded, and sectioned at 5 μm. Sections were stained with hematoxylin and eosin (H&E), and liver samples were additionally subjected to Oil Red O staining. All stained sections were examined using a digital pathology scanning system. Histological processing was outsourced to Servicebio Biotechnology Co., Ltd. (Wuhan, China).

### 2.8. Biochemical Analysis

Serum samples were analyzed for total cholesterol (TC), triglycerides (TG), low-density lipoprotein cholesterol (LDL-C), high-density lipoprotein cholesterol (HDL-C), alanine aminotransferase (ALT), aspartate aminotransferase (AST), creatinine (Cre), and blood urea nitrogen (BUN) using a Thermo Scientific Indiko Plus automated biochemical analyzer (Thermo Fisher Scientific Inc., Waltham, MA, USA). Concurrently, LPS concentrations in serum were determined by ELISA following the kit protocol.

For hepatic analyses, approximately 0.1 g of liver tissue was homogenized in ice-cold PBS (1:9, *w*/*v*), centrifuged, and the supernatant used to determine TC and TG levels with commercial kits according to the manufacturer’s protocols.

### 2.9. Colonic Short-Chain Fatty Acids (SCFAs) Analysis

Immediately after euthanasia, colonic contents were aseptically collected into sterile cryogenic tubes, snap-frozen in liquid nitrogen, and stored at −80 °C until SCFA analysis. For each sample, approximately 100 mg of colonic contents was weighed and homogenized in 1 mL of ice-cold PBS containing sterile glass beads. Bead-assisted homogenization was used to improve mechanical disruption and ensure uniform processing of the viscous colonic matrix. The homogenate was centrifuged at 5000 rpm for 10 min at 4 °C, and 700 μL of the supernatant was mixed with 175 μL of 20% (*w*/*v*) metaphosphoric acid to precipitate proteins and stabilize SCFAs. A 700 μL aliquot of the acidified supernatant was mixed with 100 μL of sodium 3-(trimethylsilyl) propionate (TSP, 0.2 mg/mL) as an internal standard and transferred to a 5-mm NMR tube. SCFAs were quantified using a 500 MHz NMR spectrometer (Bruker BioSpin AG, Fällanden, Switzerland) by comparing characteristic SCFA resonance peaks with the internal standard. All samples were processed under the same extraction, acidification, and acquisition conditions to ensure comparability. Data were processed and analyzed with R software (version 4.4.1, R Core Team, Vienna, Austria).

### 2.10. Fecal Total Bile Acids and Free Fatty Acids Analysis

Mouse fecal samples were air-dried, ground, and used for bile acids and free fatty acids extraction. For bile acids, fecal powder was mixed with 75% ethanol (1:35, *w*/*v*), incubated at 55 °C for 5 h, and centrifuged (8000 rpm, 10 min); the supernatant was collected. For free fatty acids, approximately 0.1 g of fecal powder was homogenized with 1 mL of extraction solvent (chloroform:n-heptane:anhydrous methanol = 28:21:1), shaken on ice for 10 min, and centrifuged (8000 rpm, 5 min); the supernatant was collected. Fecal total bile acids (TBA) and free fatty acids (FFA) levels were quantified using commercial kits according to the manufacturers’ instructions.

### 2.11. 16S rRNA Sequencing Analysis of Fecal Microbiota

Total microbial DNA was extracted from fecal samples according to the instructions of the FastPure Stool DNA Isolation Kit. The V3-V4 hypervariable regions of the bacterial 16S rRNA gene were PCR-amplified using primers 338F (5′-ACTCCTACGGGAGGCAGCAG-3′) and 806R (5′-GGACTACHVGGGTWTCTAAT-3′) with sample-specific barcode sequences. The PCR products were subsequently purified and quantified. Sequencing libraries were prepared through the NEXTFLEX Rapid DNA-Seq Kit, followed by sequencing on the NextSeq 2000 platform (Illumina, San Diego, CA, USA). All sequencing procedures were performed by Shanghai Majorbio Bio-pharm Technology Co., Ltd. (Shanghai, China).

### 2.12. Serum Untargeted Metabolomics Analysis

First, 100 μL of serum was mixed with 400 μL of extraction solvent (acetonitrile:methanol = 1:1, *v*/*v*) containing 0.02 mg/mL L-2-chlorophenylalanine as an internal standard. The mixture was vortexed for 30 s, followed by sonication at 5 °C and 40 kHz for 30 min. Samples were incubated at −20 °C for 30 min to precipitate proteins, centrifuged at 13,000× *g* for 15 min at 4 °C, and the resulting supernatant was collected and dried under a nitrogen stream. The dried residue was redissolved in 100 μL of solvent (acetonitrile:water = 1:1, *v*/*v*), sonicated for 5 min, and centrifuged at 13,000× *g* for 10 min. The supernatant was transferred to autosampler vials for LC-MS/MS analysis. Chromatographic separation was performed using an ultra-high-performance liquid chromatography system coupled to a tandem mass spectrometer. Both positive and negative electrospray ionization modes were used to broaden metabolite coverage. A pooled quality control sample was prepared by mixing equal aliquots from all serum samples and was injected at regular intervals throughout the analytical sequence to monitor instrument stability and analytical reproducibility. Metabolic features with poor reproducibility in QC samples were removed before downstream multivariate and pathway analyses.

### 2.13. Statistical Analysis

All data are presented as mean ± standard deviation (SD). Statistical analyses were conducted using SPSS 26.0 (SPSS Inc., Chicago, IL, USA). Before one-way ANOVA, data normality and homogeneity of variance were assessed using the Shapiro–Wilk test and Levene’s test, respectively. For data meeting these assumptions, group differences were evaluated by one-way ANOVA followed by Fisher’s LSD post hoc test. When these assumptions were not satisfied, appropriate non-parametric analysis was performed. A value of *p* < 0.05 was considered statistically significant. Graphs were generated using Origin 2022.

## 3. Results

### 3.1. Chemical Composition and Structural Features of UMP

Before evaluating the biological activity of UMP in vivo, it was necessary to determine whether ultrafiltration could effectively enrich the albumin-type protein fraction from mulberry leaves. Therefore, the chemical composition, molecular weight distribution, and secondary structure of UMP were first characterized. Based on the total protein content in mulberry leaf powder, the extraction yield of mulberry leaf protein was 30.50%. Composition analysis showed that UMP was highly enriched in protein, reaching 87.12 ± 0.52 g/100 g, while the contents of polyphenols and polysaccharides were 2.52 ± 0.00 g/100 g GAE and 8.21 ± 1.49 g/100 g, respectively (Table 1). These results indicate that ultrafiltration effectively enriched the protein fraction of mulberry leaves, although minor amounts of non-protein bioactive components, including polyphenols and polysaccharides, remained in the final UMP preparation. Therefore, UMP should be regarded as a protein-rich albumin-type fraction rather than an absolutely purified protein isolate.

**Figure 1:**
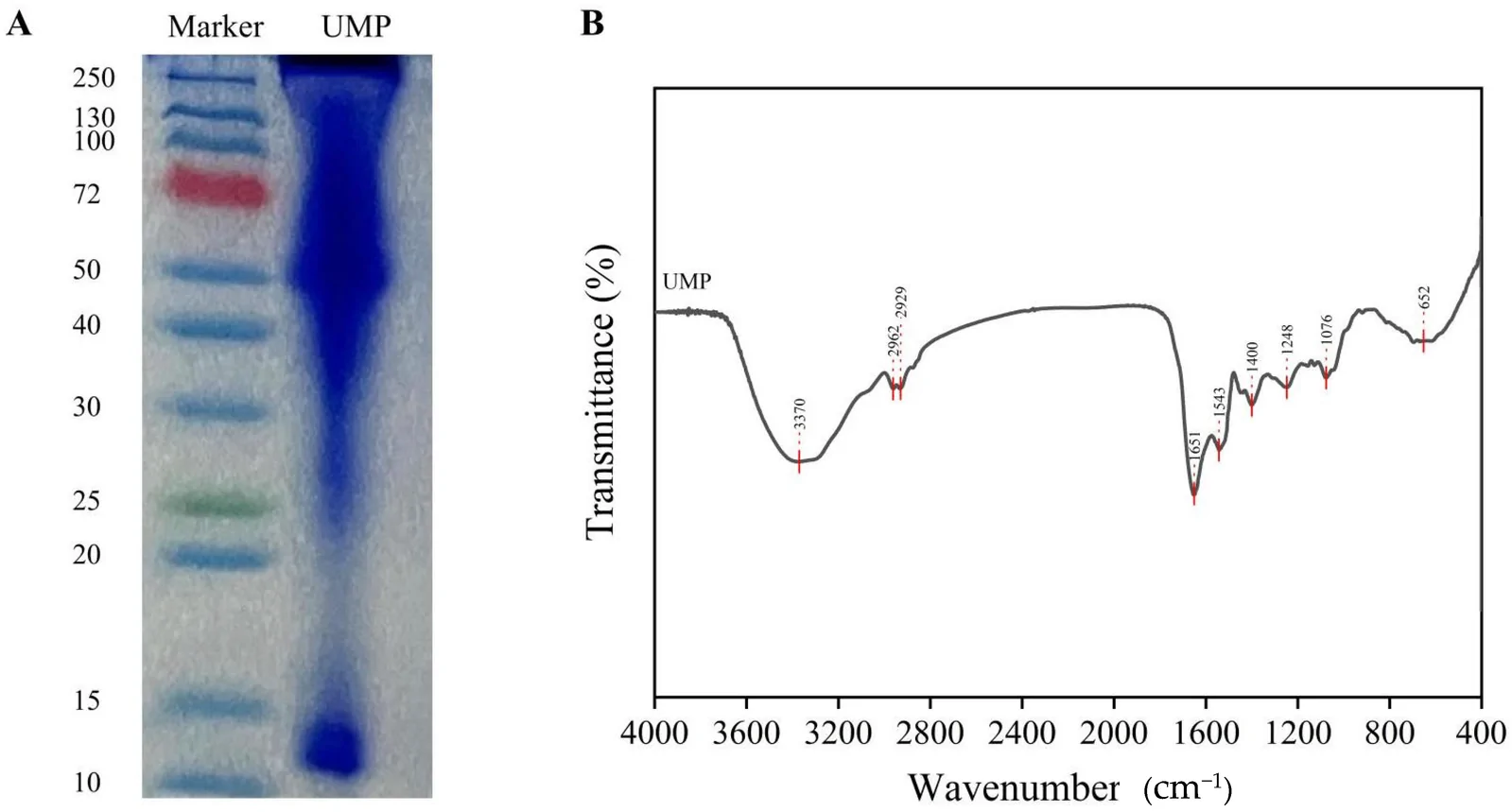
Structural characterization of the UMP. (**A**) SDS-PAGE electrophoresis analysis; (**B**) secondary structure analysis of UMP.

SDS-PAGE analysis revealed two major protein bands with approximate molecular weights of 14 kDa and 52 kDa (Figure 1A), suggesting that UMP mainly consisted of albumin-like subunits. In addition to the two major bands at approximately 14 and 52 kDa, a continuous staining signal was observed between and around the major bands. This pattern may reflect protein microheterogeneity, partial aggregation, or limited degradation during extraction and acid precipitation. Nevertheless, the dominant bands indicate that UMP was mainly enriched in albumin-like protein subunits. FTIR analysis was then performed to further characterize its structural properties. Deconvolution of the amide I region showed that UMP was predominantly composed of *β*-turns (42.26%), followed by *α*-helices (25.21%), random coils (19.89%), and *β*-sheets (12.64%) (Figure 1B and Table 2). These results confirm that UMP is a protein-rich albumin-type fraction with a distinct secondary structure. However, because minor amounts of polyphenols and polysaccharides remained in the final preparation, the biological effects observed in vivo should be interpreted as the effects of the UMP preparation as a whole rather than those of a completely purified protein alone.

### 3.2. UMP Attenuated HFD-Induced Body Weight Gain Independent of Food Intake

**Figure 2:**
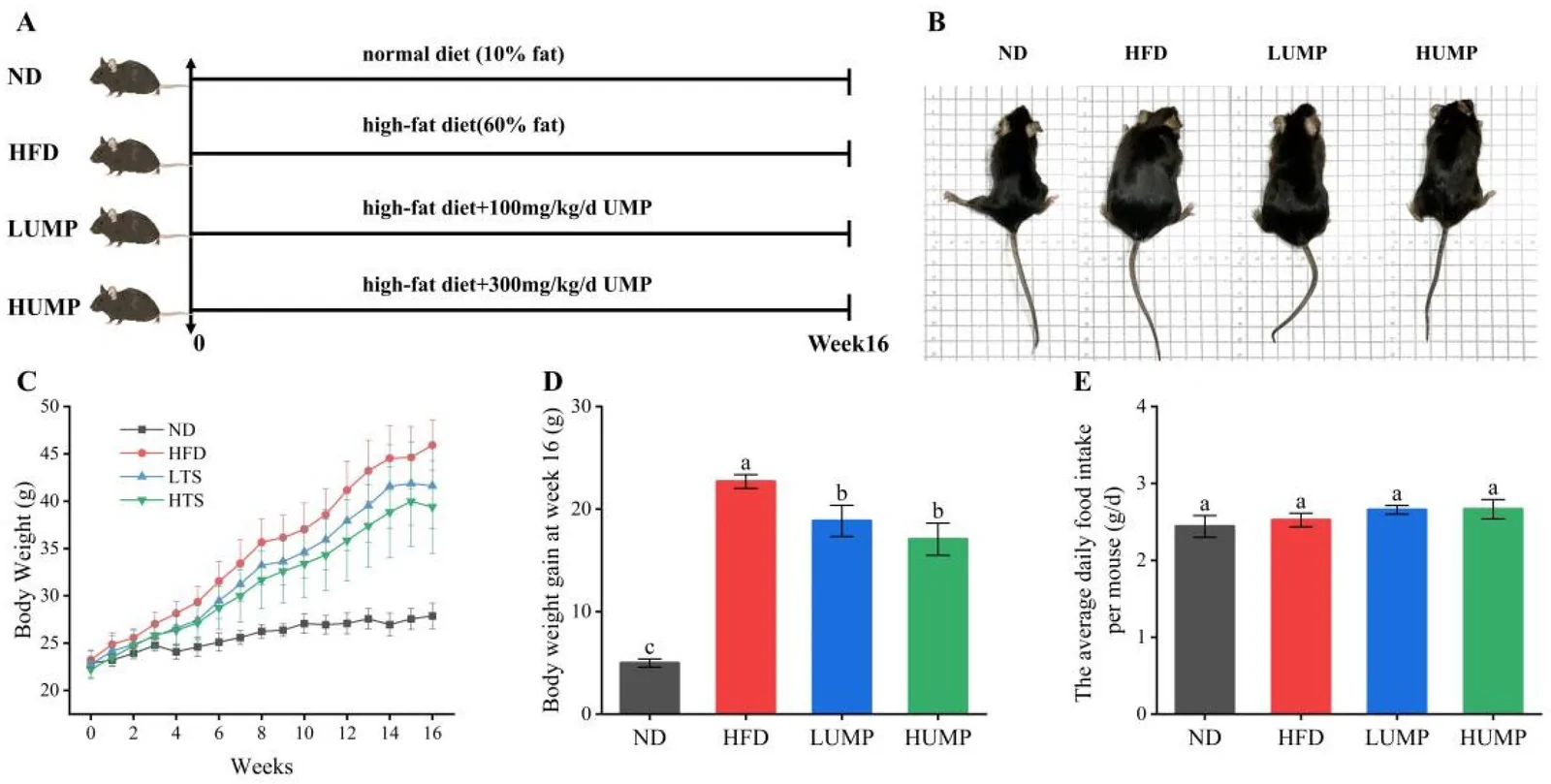
Effects of the UMP on body weight and food intake in mice. (**A**) Animal experiment design; (**B**) Representative body morphology; (**C**) weekly body weight changes; (**D**) total body weight gain; (**E**) daily food intake. Different letters indicate significant differences among groups (*p* < 0.05).

Excessive body weight gain is the most direct phenotype of diet-induced obesity. To determine whether UMP has anti-obesity activity in vivo, a 16-week HFD-induced obesity mouse model was established, and body weight changes were monitored throughout the intervention period (Figure 2A). As shown in Figure 2B–D, the body weight of HFD mice increased significantly (*p* < 0.05), whereas UMP treatment attenuated this weight gain in a dose-dependent manner. All groups had comparable body weights at week 0. After 16 weeks, weight gain of mice in the HFD group (22.69 ± 1.78 g) was 4.5-fold greater than those in the normal diet (ND) group (4.99 ± 1.06 g), demonstrating that the HFD-induced obesity model was well established (Figure 2C). Compared to the HFD group, mice in the LUMP and HUMP groups exhibited significant reductions in body weight of 3.85 g and 5.63 g, respectively (Figure 2D, *p* < 0.05).

The monitoring of food intake is critical to exclude the confounding effect of reduced caloric intake on body weight. As shown in Figure 2E, no significant differences were observed in average daily food intake among the HFD group (2.52 ± 0.13 g/d), the ND group (2.44 ± 0.20 g/d), and the UMP-treated groups (LUMP: 2.66 ± 0.08 g/d; HUMP: 2.66 ± 0.18 g/d). To further evaluate whether the reduction in body weight gain was independent of food intake, food efficiency ratio (FER) was calculated as total body weight gain divided by cumulative food intake during the 16-week intervention. Compared with the HFD group, both UMP-treated groups showed lower FER values, indicating that UMP reduced body weight gain without reducing food intake and may improve metabolic efficiency under HFD conditions. FER = total body weight gain (g)/cumulative food intake (g).

### 3.3. The UMP Improved Glucose Tolerance and Insulin Sensitivity in the HFD-Fed Mice

**Figure 3:**
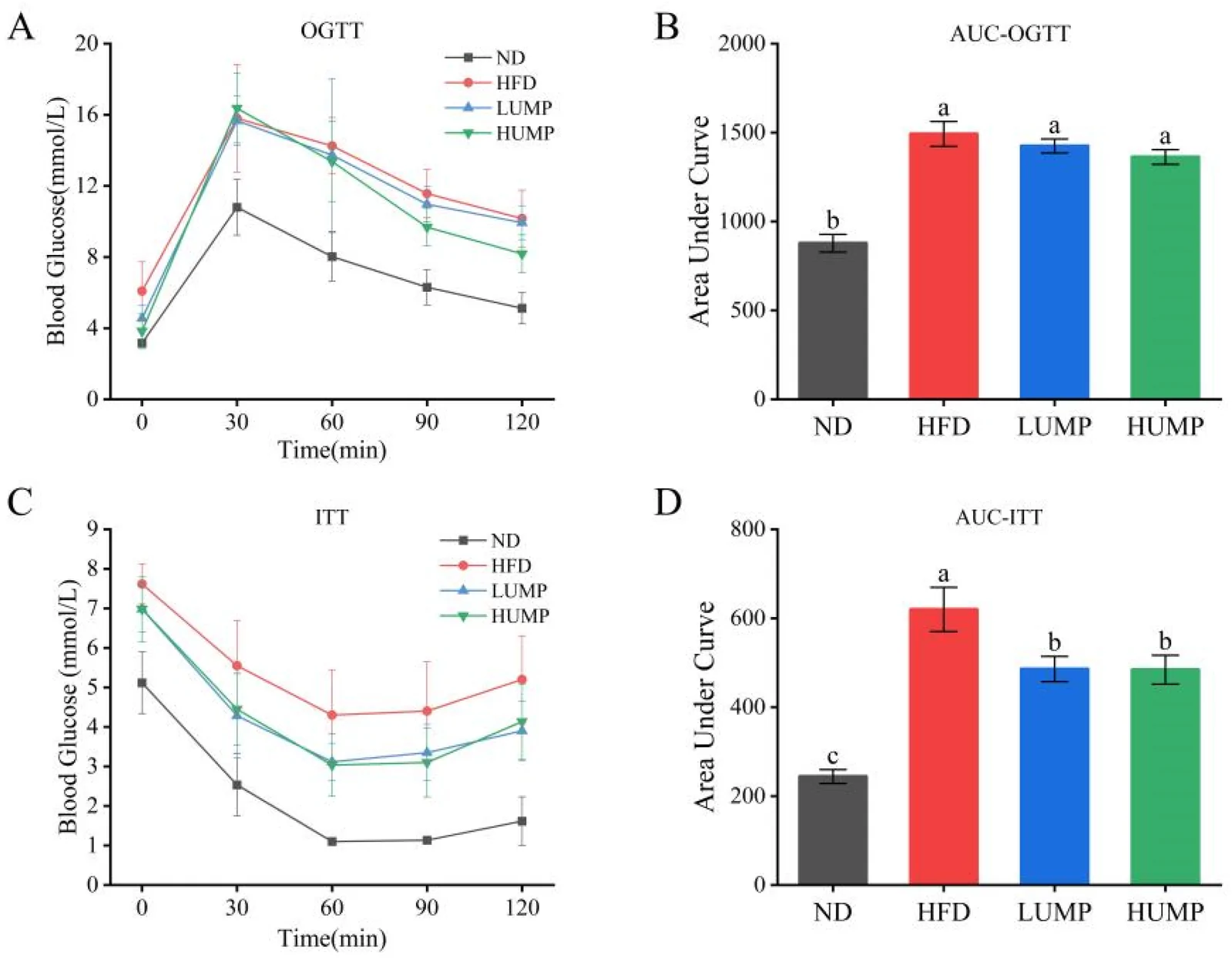
Effects of the UMP on oral glucose tolerance and insulin resistance in mice. (**A**) Oral glucose tolerance test (OGTT); (**B**) area under the OGTT curve (AUC-OGTT); (**C**) insulin tolerance test (ITT); (**D**) area under the ITT curve (AUC-ITT). Different letters indicate significant differences among groups (*p* < 0.05).

Obesity is commonly accompanied by impaired glucose utilization and insulin resistance. To determine whether UMP could improve systemic glucose homeostasis, OGTT and ITT were performed during the late stage of the intervention. Long-term HFD feeding impaired glucose tolerance, as reflected by higher blood glucose levels after glucose loading and increased OGTT-AUC values compared with the ND group (Figure 3A,B). UMP treatment did not completely normalize the OGTT curve; however, the HUMP group showed a lower blood glucose level at 60 min after glucose administration, and the AUC values decreased in a dose-dependent manner.

ITT analysis further showed that UMP improved insulin responsiveness. After insulin injection, blood glucose levels in both UMP-treated groups declined more effectively than those in HFD controls, particularly at 30, 60, and 90 min (Figure 3C). Consistently, ITT-AUC values were significantly reduced by 21.62% and 21.86% in the LUMP and HUMP groups, respectively (Figure 3D). These findings indicate that UMP partially restores glucose metabolic control and improves insulin sensitivity in HFD-fed mice.

### 3.4. The UMP Alleviated HFD-Induced Dyslipidemia and Systemic Metabolic Injury

Dyslipidemia, liver injury, metabolic endotoxemia, and renal metabolic disturbance are important systemic manifestations of obesity. Therefore, serum biochemical parameters were measured to evaluate whether UMP could improve HFD-induced metabolic injury beyond body weight control. Compared with the ND group, HFD-fed mice exhibited significantly increased serum TG, TC, LDL-C, ALT, AST, Cre, and LPS levels, together with reduced BUN levels (Table 3). These changes indicate that long-term HFD feeding caused lipid metabolic disorder, hepatic injury, metabolic inflammation, and renal metabolic disturbance. UMP supplementation significantly decreased serum TG, TC, LDL-C, ALT, Cre, and LPS levels, with the high-dose treatment generally showing stronger effects. AST levels also showed a downward trend after UMP treatment.

Notably, HDL-C levels were not significantly changed among groups, suggesting that UMP mainly improved pathological lipid accumulation rather than broadly altering all circulating lipoproteins. In addition, partial restoration of BUN levels in the HUMP group might reflect improved nitrogen metabolism and renal-related metabolic status. Overall, these results demonstrate that UMP ameliorates HFD-induced dyslipidemia and systemic metabolic injury.

### 3.5. The UMP Reduced Hepatic Lipid Accumulation and Protected Against HFD-Induced Liver Injury

**Figure 4:**
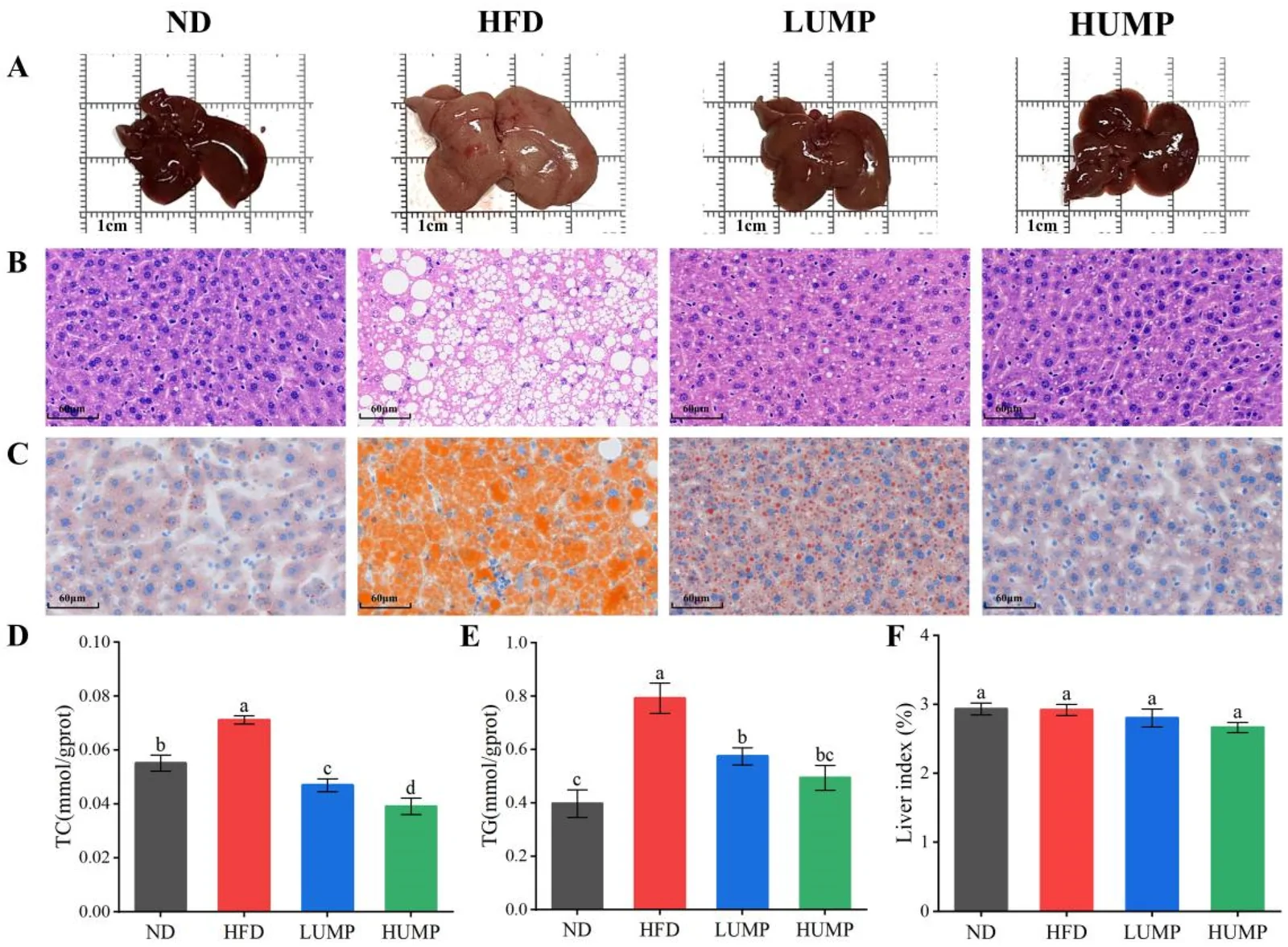
Effects of the UMP on liver injury in mice. (**A**) Representative images of livers from each group; (**B**) H&E staining (40×); (**C**) Oil Red O staining (40×); (**D**) Hepatic TC content; (**E**) Hepatic TG content; (**F**) Liver index. Different letters indicate significant differences among groups (*p* < 0.05).

The liver is the central organ for lipid metabolism, and hepatic steatosis is a major pathological consequence of HFD-induced obesity. Therefore, liver morphology, histopathology, and hepatic lipid contents were examined to determine whether UMP could directly protect against obesity-associated liver injury. Gross observation showed that livers from HFD-fed mice were enlarged and pale, which is typical of hepatic lipid accumulation (Figure 4A). In contrast, livers from UMP-treated mice showed improved color and morphology. H&E staining further revealed severe lipid vacuolation and inflammatory infiltration in the HFD group, whereas UMP treatment markedly reduced these pathological changes, especially in the HUMP group (Figure 4B). Oil Red O staining confirmed that HFD induced extensive hepatic lipid droplet deposition, while UMP supplementation substantially decreased lipid accumulation (Figure 4C).

Consistent with the histological observations, HFD feeding significantly increased hepatic TC and TG levels. UMP treatment reduced both hepatic TC and TG contents in a dose-dependent manner (Figure 4D,E). The liver index showed no marked difference among groups (Figure 4F), indicating that UMP reduced hepatic lipid deposition without adversely affecting liver growth. These results suggest that UMP exerts a clear hepatoprotective effect by suppressing HFD-induced hepatic steatosis.

### 3.6. The UMP Protected Colonic Morphology and Increased Colonic SCFA Production

**Figure 5:**
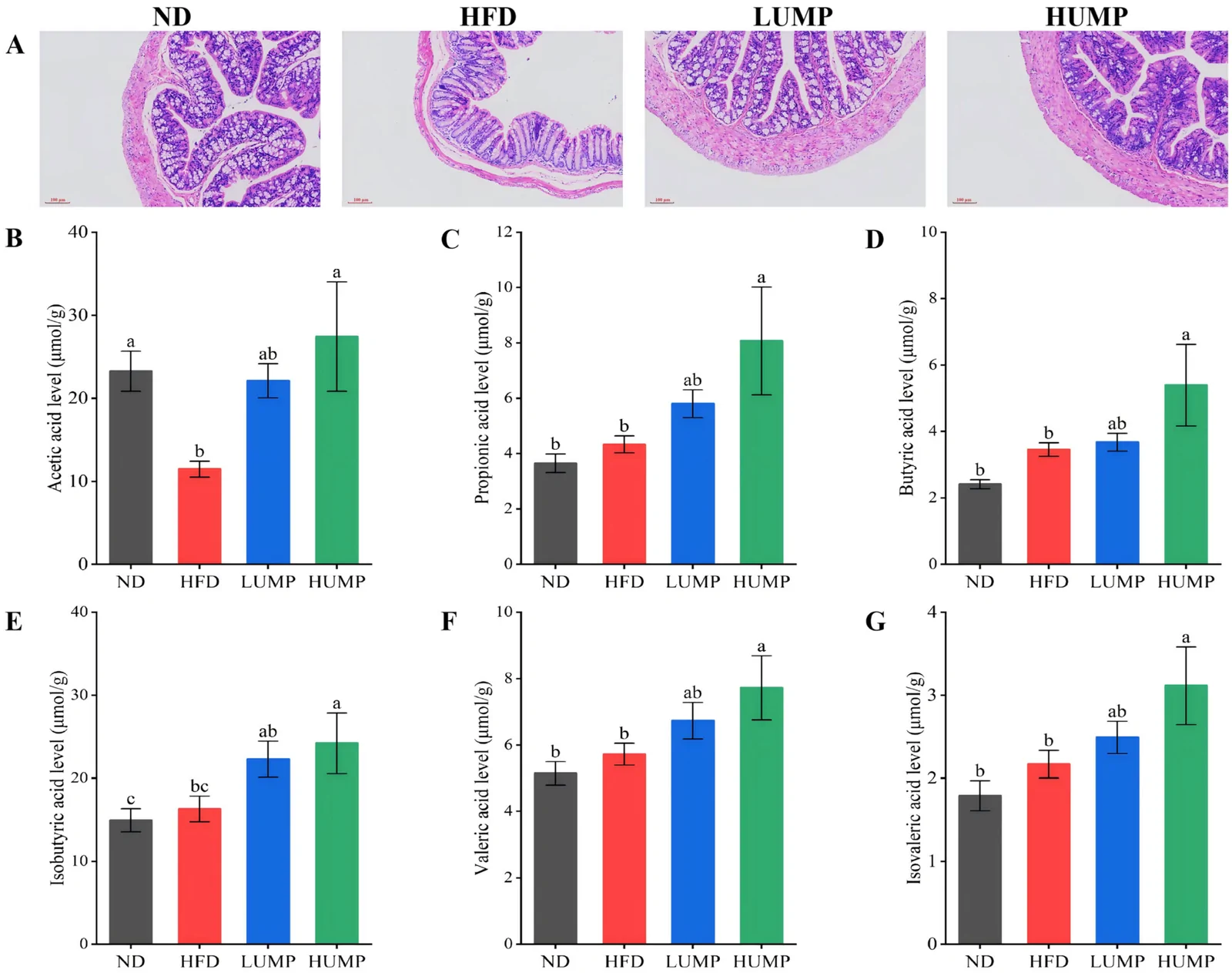
Effects of UMP on colonic morphology and SCFAs levels in mice. (**A**) H&E staining (12×); (**B**–**G**) concentrations of short-chain fatty acids (acetate, propionate, butyrate, isobutyrate, valerate, and isovalerate). Data are presented as mean ± SD. Different lowercase letters indicate statistically significant differences among groups based on one-way ANOVA followed by Fisher’s LSD post hoc test (*p* < 0.05). Groups sharing the same letter are not significantly different.

The colon is the main site of gut microbial fermentation and plays an essential role in maintaining intestinal barrier function. Since obesity-related metabolic disorders are closely linked to intestinal injury and microbial metabolite imbalance, colonic morphology and short-chain fatty acid levels were analyzed. H&E staining showed that ND mice had intact colonic architecture, well-organized crypts, and abundant goblet cells (Figure 5A). In contrast, HFD feeding caused obvious colonic injury, including crypt disruption, reduced goblet cell abundance, inflammatory cell infiltration, and thinning of the muscularis externa. UMP treatment alleviated these pathological changes in a dose-dependent manner. In particular, the HUMP group showed improved crypt integrity, increased goblet cell numbers, reduced inflammatory infiltration, and a more preserved colonic structure.

Because SCFAs are key microbial metabolites involved in intestinal barrier maintenance, anti-inflammatory regulation, and host energy metabolism, six major SCFAs were quantified in colonic contents. Compared with the HFD group, UMP treatment markedly increased the levels of acetate, propionate, butyrate, isobutyrate, valerate, and isovalerate, with the most prominent increases observed in the HUMP group (Figure 5B–G). These findings suggest that UMP improves intestinal health not only by preserving colonic morphology but also by enhancing microbial fermentation-derived SCFA production.

### 3.7. The UMP Modulates Fecaled Total Bile Acids (TBA) and Free Fatty Acids (FAS) Profiles

**Figure 6:**
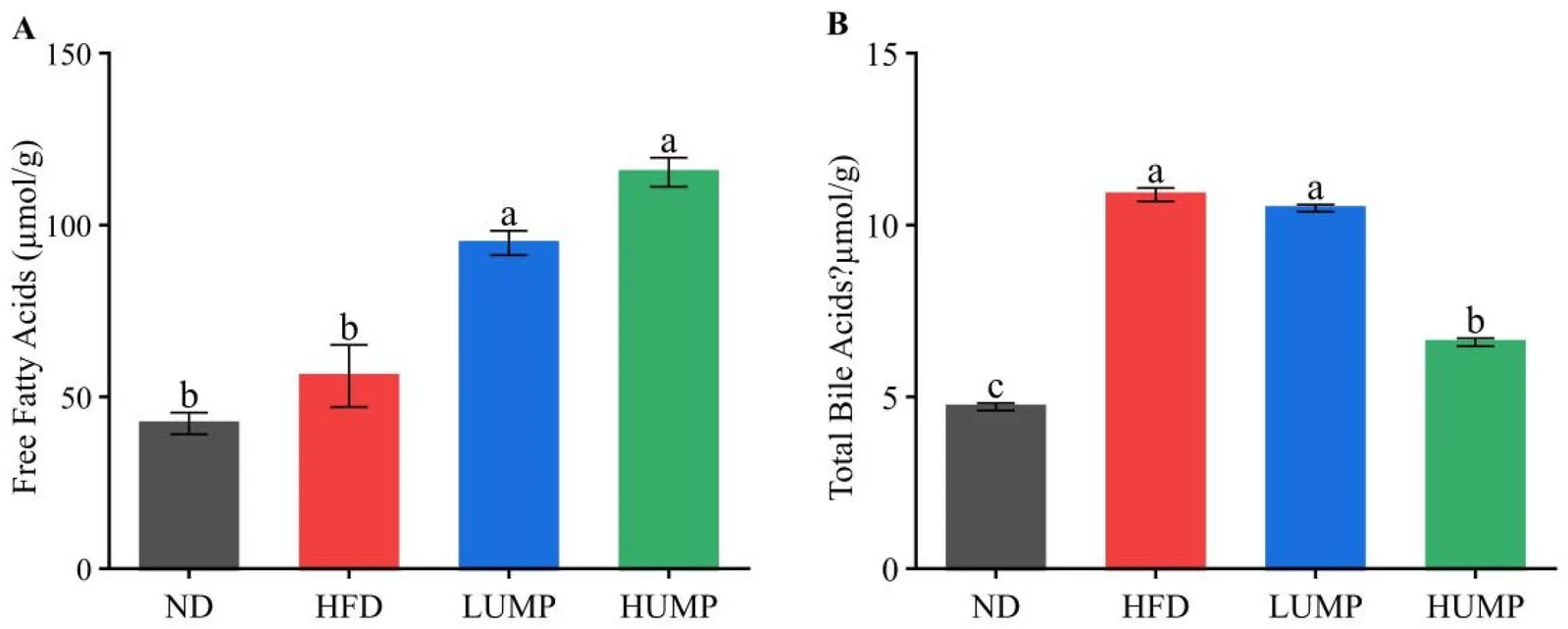
Effects of UMP on fecal total bile acids and free fatty acids. (**A**) Free fatty acids levels; (**B**) total bile acids levels. Different letters indicate significant differences among groups (*p* < 0.05).

Fecal free fatty acids reflect intestinal lipid handling, whereas fecal total bile acids are closely associated with bile acid circulation and lipid digestion. To further explore how UMP affects intestinal lipid metabolism, fecal FFA and TBA levels were measured. As shown in Figure 6A, fecal FFAs were slightly increased in HFD mice compared with the ND group, but the difference was not statistically significant. In contrast, UMP treatment markedly elevated fecal FFAs levels in a dose-dependent manner (*p* < 0.05). This indicated that UMP reduces intestinal fatty acids absorption, thereby limiting lipid accumulation and metabolic stress in the liver and pancreas.

Bile acids play a central role in lipid digestion and metabolic regulation via the farnesoid X receptor (FXR) and TGR5 signaling pathways. Figure 6B showed that fecal TBA levels were markedly elevated in HFD mice compared with the ND group, indicating disrupted bile acid enterohepatic circulation. The UMP supplementation significantly reduced fecal TBA levels, particularly in the HUMP group, although levels remained higher than those in ND controls. These results indicate that UMP partially restores HFD-disrupted bile acid metabolism, which may contribute to improved lipid metabolism and gut microbial balance.

### 3.8. The UMP Restored Gut Microbiota Diversity and Reshaped Microbial Composition

**Figure 7:**
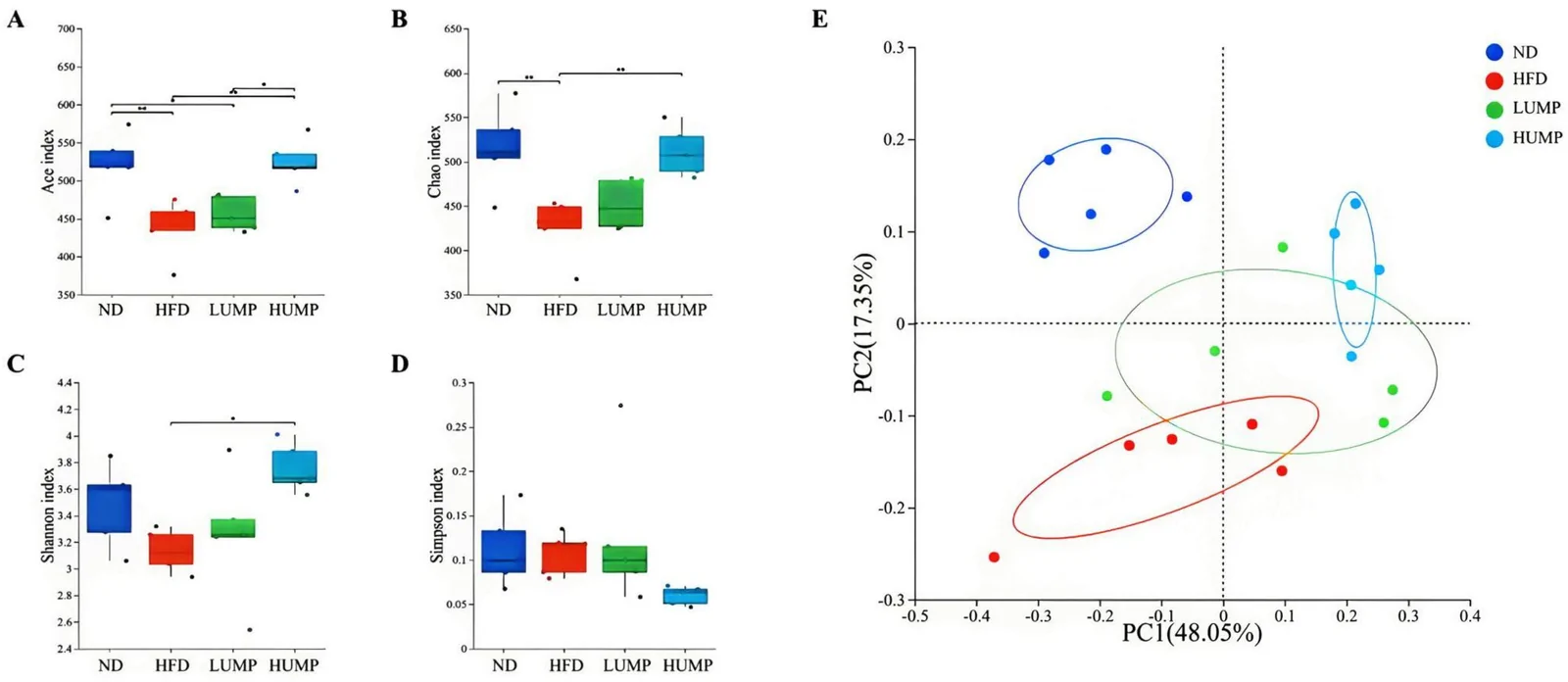
Gut microbiota diversity analysis. (**A**) ACE index; (**B**) Chao index; (**C**) Shannon index; (**D**) Simpson index; (**E**) Principal component analysis (PCA) based on genus-level composition. The circles represent individual samples.

Gut microbiota dysbiosis is a key contributor to HFD-induced obesity, metabolic inflammation, and impaired energy homeostasis. Therefore, 16S rRNA sequencing was performed to determine whether the metabolic benefits of UMP were accompanied by remodeling of gut microbial communities. Alpha-diversity analysis showed that HFD feeding significantly reduced the Ace and Chao indices and also decreased the Shannon index, while increasing the Simpson index (Figure 7A–D). These changes indicate that HFD reduced microbial richness and diversity. UMP treatment reversed these alterations in a dose-dependent manner, with the HUMP group showing the strongest restoration of *α*-diversity.

Beta-diversity analysis based on Bray–Curtis distance further showed clear separation between ND and HFD groups, indicating that HFD markedly altered the overall gut microbial community structure (Figure 7E). UMP-treated samples clustered closer to the ND group, suggesting partial recovery of the gut microbiota profile. Together, these findings indicate that the UMP preparation, a protein-rich albumin-type fraction containing minor amounts of polyphenols and polysaccharides, attenuates HFD-induced obesity in association with coordinated modulation of gut microbiota, intestinal metabolites, and systemic metabolic homeostasis.

**Figure 8:**
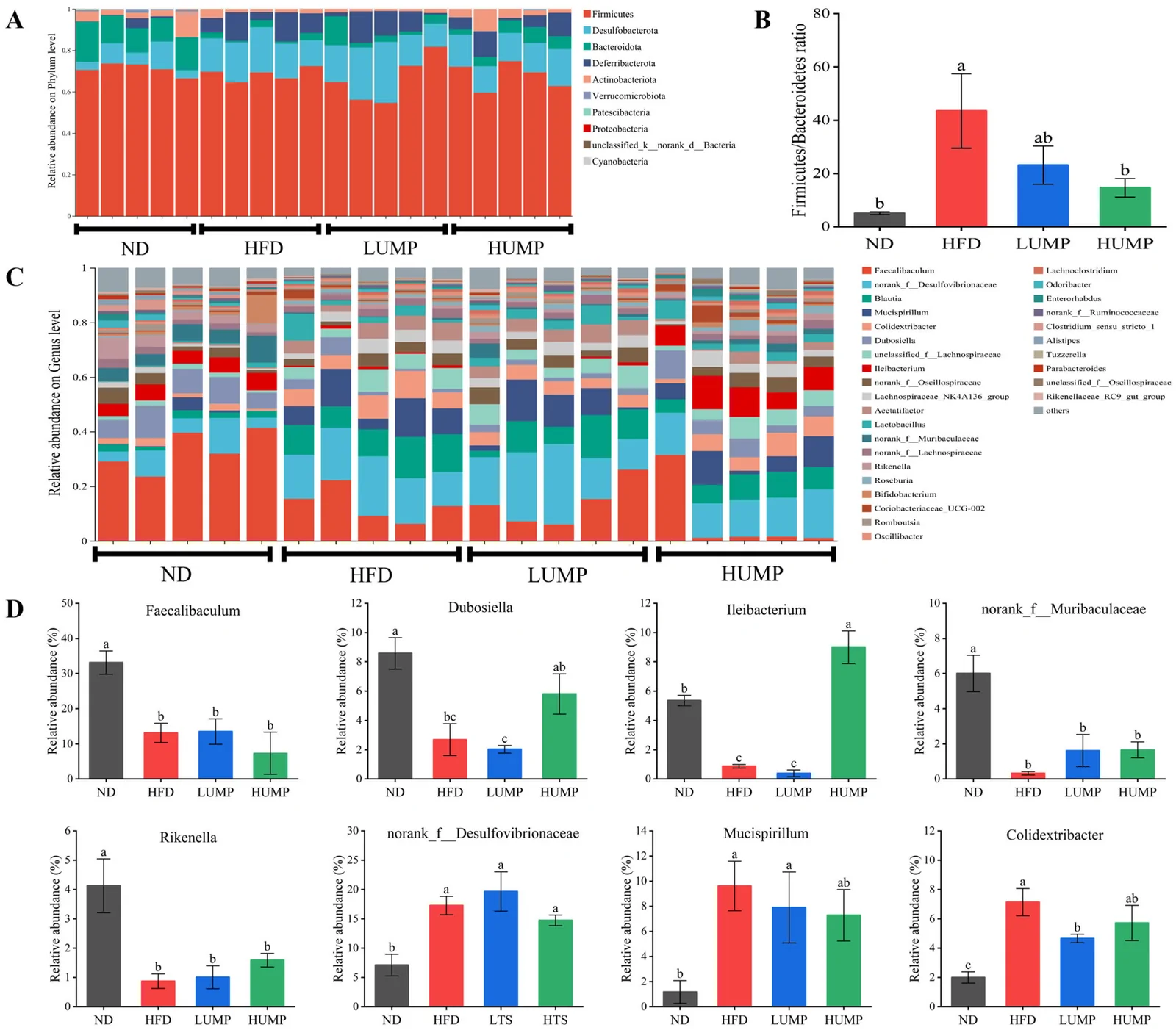
UMP-mediated shifts in gut microbiota composition at the phylum and genus levels. (**A**) Stacked bar plots showing relative abundance at the phylum level; (**B**) Firmicutes/Bacteroidota (F/B) ratio; (**C**) Stacked bar plots showing relative abundance at the genus level; (**D**) Relative abundance of key genera. Different letters indicate significant differences among groups (*p* < 0.05).

To further identify the taxa involved in UMP-mediated microbiota remodeling, microbial composition was analyzed at both phylum and genus levels. At the phylum level (Figure 8A), Firmicutes, Bacteroidota, Desulfobacterota, Deferribacterota, and Actinobacteriota were the dominant taxa in mouse gut microbiota, with Firmicutes being the most abundant. HFD feeding markedly reduced the relative abundance of Bacteroidota, Actinobacteriota, and Verrucomicrobiota, while increasing Desulfobacterota and Deferribacterota, indicating significant dysbiosis. These results are consistent with previous reports. UMP supplementation, particularly at high dose, partially restored the relative abundances of beneficial phyla. Consistently, the Firmicutes/Bacteroidota (F/B) ratio (Figure 8B) increased nearly nine-fold in HFD mice (from 5.13 to 43.5) but was significantly reduced by UMP (23.1 in LUMP, 14.6 in HUMP), suggesting improved microbial balance.

At the genus level, HFD markedly depleted *Faecalibaculum*, *Dubosiella*, *Ileibacterium*, *norank_f_Muribaculaceae*, and *Rikenella*, while enriching *norank_f_Desulfovibrionaceae*, *Blautia*, *Mucispirillum*, and *Colidextribacter* (Figure 8C). UMP intervention reversed most of these changes in a dose-dependent manner, significantly increased the relative abundance of *Dubosiella*, *Ileibacterium*, *norank_f_Muribaculaceae*, and *Rikenella*, and reduced the abundance of *norank_f_Desulfovibrionaceae*, *Mucispirillum*, and *Colidextribacter* (Figure 8D). Notably, *Faecalibaculum* remained low despite UMP treatment, suggesting that UMP modulates specific subsets of beneficial bacteria rather than a broad spectrum. Together, these results indicate that UMP reshapes gut microbial structure by promoting beneficial genera and suppressing potentially pathogenic ones, thereby mitigating HFD-induced dysbiosis.

### 3.9. The UMP Reshaped Serum Metabolic Profiles in HFD-Fed Mice

**Figure 9:**
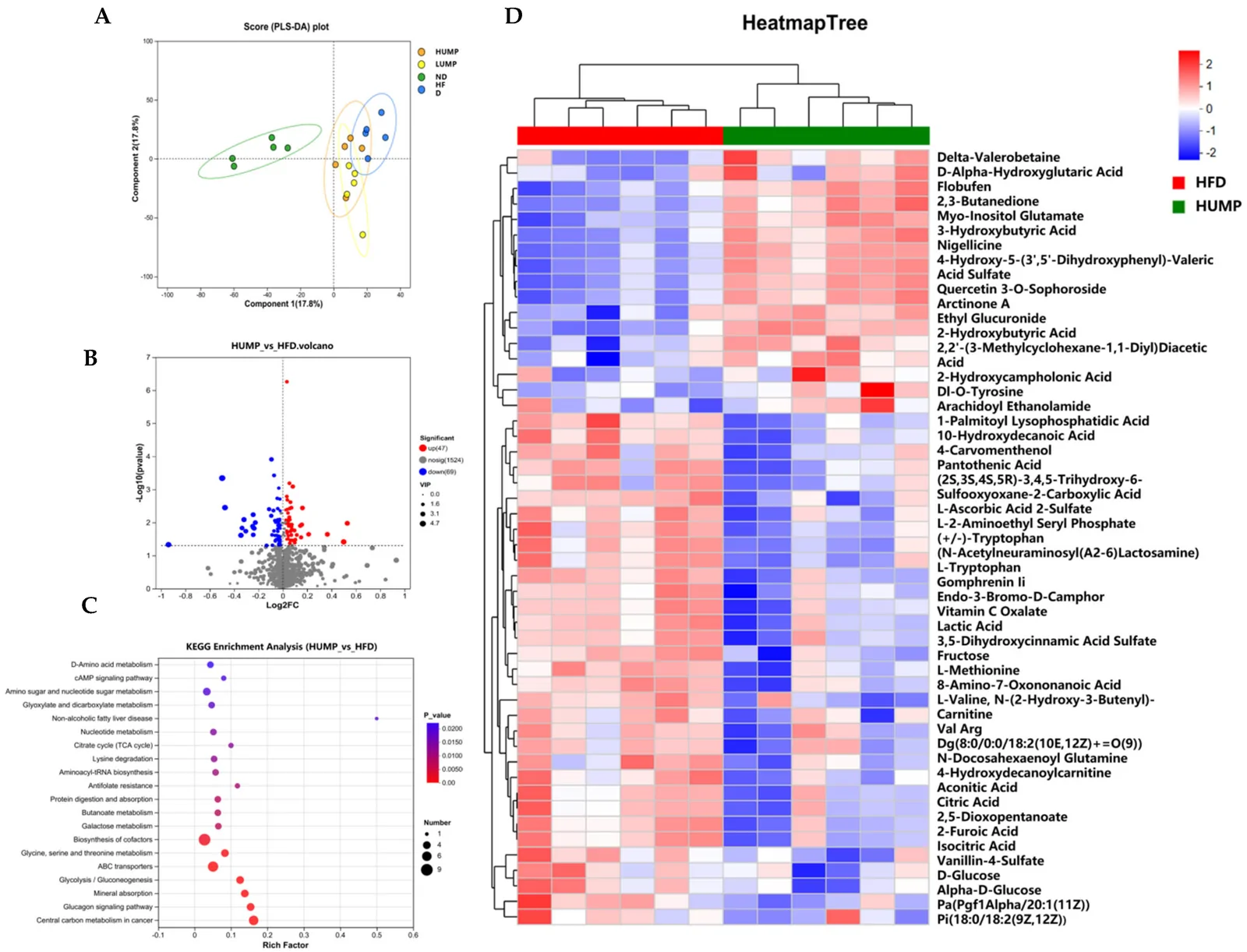
Effects of UMP on serum metabolomic profiles in mice. (**A**) PLS-DA score plot of serum metabolites; (**B**) Volcano plot of differential metabolites; (**C**) KEGG-based prediction of differential serum metabolic pathways; (**D**) Heatmap showing relative abundance of significantly altered metabolites. The circles represent individual samples in the (**A**).

Because gut microbiota-derived signals can influence systemic metabolism, untargeted serum metabolomics was performed to determine whether UMP-induced microbial remodeling was accompanied by host metabolic reprogramming. Partial least squares discriminant analysis (PLS-DA) revealed distinct clustering among groups (R^2^X = 0.468, R^2^Y = 0.996, Q^2^ = 0.671), with a clear separation between ND and HFD mice, indicating marked metabolic alterations induced by HFD (Figure 9A). Both LUMP and HUMP groups shifted toward the ND cluster, suggesting that UMP supplementation mitigates HFD-induced metabolic disturbances.

Differential metabolites between the HFD and HUMP groups were screened using OPLS-DA (VIP > 1, *p* < 0.05) and visualized by a volcano plot (Figure 9B). Hierarchical clustering of the top 50 metabolites further illustrated the distinct metabolic patterns between groups, with UMP reversing HFD-induced changes in lipid, amino acid, and bile acid metabolites (Figure 9D). In total, 116 metabolites were significantly altered by HUMP treatment, including 47 upregulated (e.g., 7-ketolithocholic acid, arachidonic acid, betaine) and 69 downregulated metabolites (e.g., D-glucose, 4-hydroxydecanoylcarnitine). These changes suggest that UMP improves glucose homeostasis, enhances lipid metabolism, and reduces excessive fatty acid oxidation, potentially via multi-target metabolic regulation.

KEGG pathway enrichment analysis identified 30 significantly modulated pathways, with the top 20 including biosynthesis of cofactors, ATP-binding cassette (ABC) transporters, glucagon signaling, glycine/serine/threonine metabolism, amino sugar and nucleotide sugar metabolism, butanoate metabolism, and protein digestion/absorption (Figure 9C). Collectively, these results indicate that UMP alleviates HFD-induced metabolic disorders through multi-target regulation of key metabolic pathways, ultimately restoring systemic metabolic homeostasis.

### 3.10. Correlation Analysis Revealed a Microbiota-Metabolite Regulatory Axis Associated with UMP Intervention

**Figure 10:**
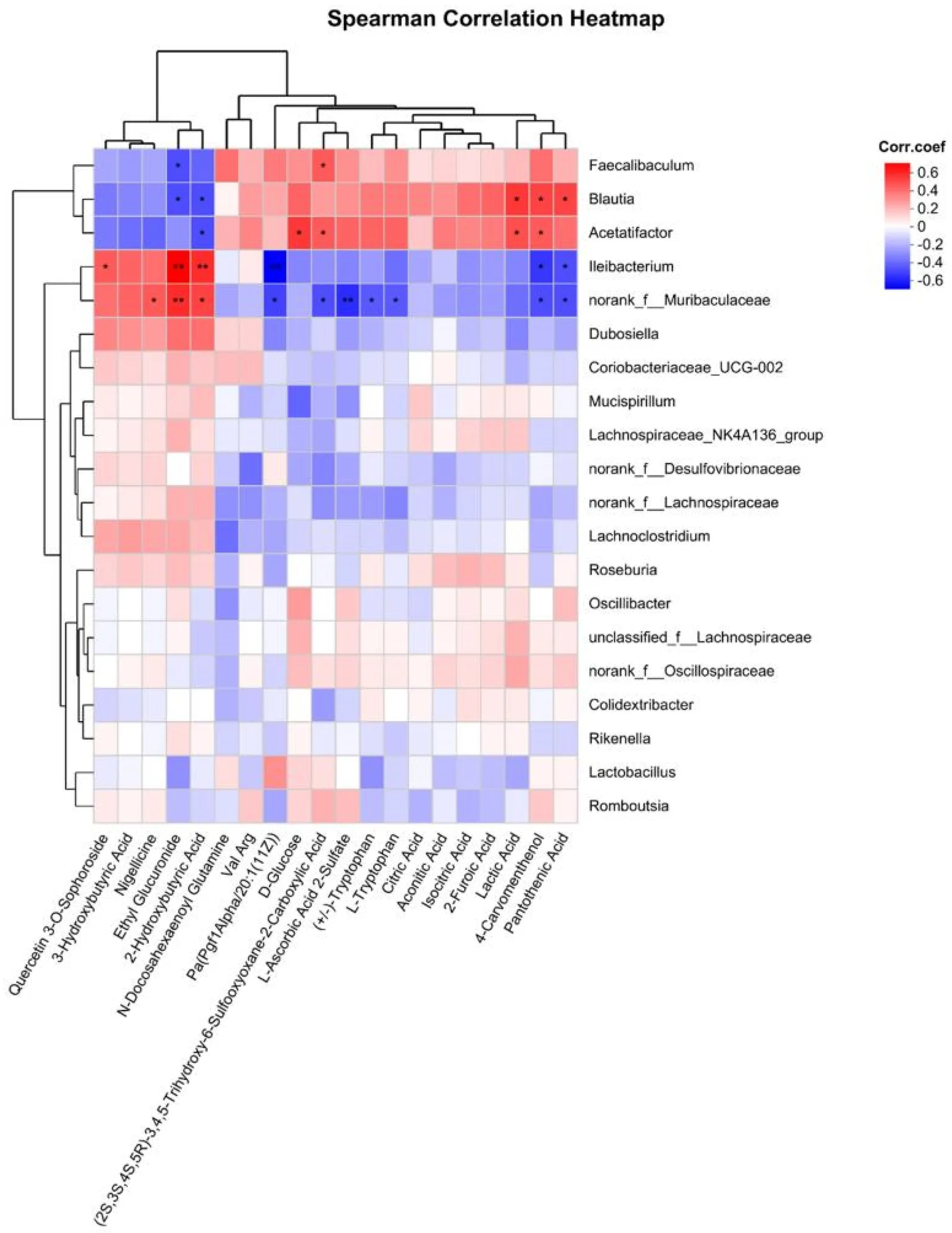
Correlation heatmap between gut microbiota and serum metabolites in mice. Red indicates positive correlations; blue indicates negative correlations; the intensity of the color reflects the strength of the correlation. Asterisks denote statistical significance: * *p* < 0.05, ** *p* < 0.01.

To further connect gut microbiota remodeling with systemic metabolic improvement, correlation analysis was performed between key bacterial taxa and differential serum metabolites. This analysis aimed to identify potential microbiota-metabolite interactions that may contribute to the anti-obesity effects of UMP (Figure 10). The *Ileibacterium* showed a positive correlation with quercetin-3-O-sophoroside, suggesting a potential link between UMP-enriched bacteria and metabolites involved in glucose and lipid metabolism. Both *Ileibacterium* and *norank*_*f*_*Muribaculaceae* were positively correlated with 2-hydroxybutyric acid, a metabolite associated with fatty acid metabolism, insulin sensitivity, and inflammatory regulation. In contrast, these genera were negatively correlated with pantothenic acid, which may reflect altered utilization of pantothenic acid for coenzyme A-related energy metabolism. These correlations suggest that UMP may act through a microbiota-metabolite-host regulatory axis. Specifically, UMP enriches beneficial bacterial taxa, promotes SCFA production and intestinal metabolic remodeling, regulates circulating metabolites involved in lipid and glucose metabolism, and ultimately improves obesity-related metabolic dysfunction.

## 4. Discussion

Rising global concerns on obesity have spurred intensive research into potential dietary strategies, with a growing focus on interventions derived from natural products. Plant proteins have emerged as a promising dietary approach for weight management and mitigating obesity, a benefit largely attributed to their nutrient density and functional properties [30,31]. Although mulberry leaf protein has been reported to possess antioxidant and anti-inflammatory activities, its efficacy in ameliorating obesity-related metabolic disorders remains to be characterized [22,23]. The present study demonstrated for the first time that ultrafiltered mulberry leaf albumin-type protein (UMP) alleviates HFD-induced obesity in mice via a microbiota-dependent, multi-pathway regulatory mechanism, highlighting UMP’s potential as a functional food ingredient.

Ultrafiltration produced a high-purity mulberry leaf albumin with a distinctive structure. The acquisition of high-quality protein samples is a prerequisite for downstream structural and functional characterization. The 10 kDa MWCO membrane was expected to retain protein subunits larger than the nominal cut-off, including the 14 kDa and 52 kDa bands observed in SDS-PAGE. However, peptides below 10 kDa may have passed through the membrane and were not included in the final UMP fraction. Therefore, although the present study focused on the retained albumin-type protein fraction, the potential activity of smaller peptides should be investigated in future fractionation studies. Ultrafiltration serves this goal effectively by concentrating the protein and removing a broad spectrum of impurities under mild physical conditions, thereby preserving the native conformation and bioactivity while maintaining high recovery yields [32,33]. Previous studies have shown that human growth hormone (hGH), a multifunctional protein hormone, contains a specific domain responsible for its lipolytic and anti-adipogenic properties [34,35]. The peptide identified within this domain is rich in *β*-turn and *α*-helix structures and retains anti-obesity activity [34]. The presence of residual polyphenols and polysaccharides is an important limitation of this study. Both classes of compounds have been reported to modulate gut microbiota and host lipid metabolism. Therefore, although the high protein content suggests that the albumin-type protein fraction may be a major contributor, the possible involvement of co-existing polyphenols and polysaccharides cannot be excluded. UMP is also abundant in these two secondary structures, suggesting that it may likewise possess anti-obesity potential. Although UMP was designed as a protein-enriched fraction, it still contained small amounts of polyphenols and polysaccharides. Therefore, the metabolic benefits observed in this study should not be interpreted as being exclusively proteogenic. Residual polyphenols may exert antioxidant and lipid-regulatory effects, whereas polysaccharides may act as fermentable substrates for gut microbiota and promote SCFA production. Thus, UMP may function as a multi-component dietary protein fraction in which albumin-type proteins act together with residual bioactive compounds to remodel gut microbiota and metabolic homeostasis. Although UMP was highly enriched in protein, minor amounts of polyphenols and polysaccharides remained in the final preparation. In addition, cellulose and hemicellulose were not directly quantified in the present study. Therefore, the observed metabolic effects cannot be attributed exclusively to the albumin-type protein fraction. Future studies using more highly purified preparations and targeted compositional analyses are required to further clarify the relative contribution of the protein fraction and co-existing non-protein components.

The anti-obesity effects of UMP are comprehensive, encompassing reduced weight gain, improved glucose tolerance and insulin sensitivity, ameliorated dyslipidemia, mitigation of hepatic steatosis, and protection of colonic tissue. Notably, UMP supplementation significantly improved a spectrum of serum biochemical parameters, which are critical markers of metabolic health and organ function. Our findings highlight UMP’s multi-organ protective effects: (1) Lipid-lowering effects: UMP’s reduction in TG and TC is comparable to that of pea albumin, and may be mediated by increased bile acid excretion (see Section 3.7) which enhances cholesterol catabolism [12]; (2) Hepatoprotective effects: Reduced ALT and AST levels indicate alleviated hepatic steatosis, confirmed by histological analysis (Section 3.5); (3) Anti-inflammatory effects: Decreased LPS levels suggest improved intestinal barrier function, which reduces chronic low-grade inflammation driving metabolic dysfunction [36]; and (4) Renal protective effects: Restored BUN and Cre levels indicate improved protein metabolism and renal excretory function, which is critical for long-term metabolic health.

Distinctively, UMP achieved pronounced reductions in body weight gain and metabolic disturbances without reducing food intake, implying a metabolic reprogramming mechanism rather than an anorectic effect. This feature distinguishes UMP from most reported protein-based interventions that rely primarily on appetite suppression or caloric restriction [30,37]. In our study, UMP exhibited multifaceted anti-obesity activities through modulation of the gut microbiota and host metabolism. The gut microbiota, a critical mediator of diet-induced obesity and metabolic dysregulation, is thus a viable intervention target. In line with earlier reports [38,39], HFD reduced α-diversity and induced a pro-obesogenic shift in the microbiota, characterized by a higher Firmicutes/Bacteroidota ratio predictive of enhanced energy harvest and inflammation. Additionally, *Norank_f__Desulfovibrionaceae* and *Mucispirillum* are notable LPS-producing pro-inflammatory taxa, whose abundance is significantly increased under HFD conditions [40]. Similarly to rice endosperm, soy and oat proteins, UMP alleviated gut microbial dysbiosis, raised the abundance of SCFA-producing taxa and improved glucose and lipid metabolic homeostasis [41,42,43]. The reduction in body weight gain without altered food intake suggests that UMP affected energy balance through mechanisms other than appetite suppression. It is consistent with reports that several plant-derived proteins, such as pea, soy, and rice-derived proteins, can improve metabolic phenotypes by modulating lipid metabolism, gut microbiota composition, and microbial metabolites rather than simply suppressing appetite [10,11]. Increased fecal FFA excretion indicates reduced intestinal lipid absorption or enhanced lipid elimination, while bile acid remodeling may influence lipid emulsification, enterohepatic circulation, and metabolic signaling through FXR/TGR5 pathways. In addition, serum metabolomics indicated changes in pathways associated with lipid turnover and glucose utilization [44]. Together, these findings suggest that UMP may reduce energy harvesting from dietary fat and improve systemic energy expenditure or substrate utilization.

The HUMP group exhibited the highest levels of acetate, propionate, and butyrate. Particularly, butyrate exerts pleiotropic beneficial effects, including enhancing the intestinal barrier through upregulation of tight junction proteins (claudin-3, claudin-4), inhibiting IL-1*β*-mediated NF-*κ*B activation, and thereby modulating intestinal immunity and inflammation [45,46,47]. The elevated SCFAs levels in UMP-treated groups likely contribute to the observed improvements in colonic morphology, reduced LPS translocation (Section 3.4), and enhanced insulin sensitivity (Section 3.3). This is consistent with previous studies showing that SCFA-producing bacteria are critical mediators of plant protein-induced metabolic benefits [42,48]. SCFAs are not merely fermentation products but also important signaling molecules linking gut microbial metabolism to intestinal barrier function and host energy homeostasis [49]. Butyrate serves as a major energy substrate for colonocytes and supports epithelial renewal, whereas acetate and propionate participate in lipid and glucose metabolism through G-protein-coupled receptor-mediated signaling [50,51]. Therefore, the elevated SCFA levels observed in the LUMP and HUMP groups may contribute to the restoration of colonic morphology, reduction in LPS translocation, and improvement of insulin sensitivity. These findings suggest that UMP-induced enrichment of SCFA-producing bacteria provides a mechanistic bridge between gut microbiota remodeling and the alleviation of HFD-induced intestinal and metabolic injury. The enrichment of *Ileibacterium* and *norank_f_Muribaculaceae* is particularly significant, as these genera are key SCFA producers [39,52], and their elevated abundance correlates with increased colonic SCFA levels (Section 3.6) and improved insulin sensitivity (Section 3.3). Moreover, the greater representation of *Dubosiella* may contribute to bile acid metabolism regulation (Section 3.7) through bile salt hydrolase (BSH) activity, linking gut microbiota to systemic metabolic improvements [53]. The enrichment of *Dubosiella* may also be relevant to bile acid remodeling. Bacteria with bile salt hydrolase activity can deconjugate taurine- or glycine-conjugated bile acids, thereby altering bile acid hydrophobicity, intestinal reabsorption, fecal excretion, and secondary bile acid generation [54,55]. Therefore, the increase in *Dubosiella* after UMP intervention may contribute to the partial normalization of bile acid metabolism by modulating enterohepatic circulation and bile acid signaling. Such microbiota-mediated metabolic benefits have been widely reported for plant-derived bioactive protein, which are capable of restoring microbial equilibrium and reinforcing intestinal tight junctions to alleviate diet-induced obesity [56,57]. Although the F/B ratio was reduced by UMP treatment, this index should be interpreted cautiously because the association between the F/B ratio and obesity is not always consistent across studies. Therefore, genus-level remodeling may provide more functionally meaningful information. In this study, UMP enriched *Ileibacterium* and *norank_f_Muribaculaceae*, which are associated with SCFA production and metabolic health, while also increasing *Dubosiella*, a genus potentially linked to bile acid transformation. These genus-level changes may be more directly related to the observed improvements in gut barrier function, lipid metabolism, and systemic metabolic homeostasis than phylum-level shifts alone. Although gut microbiota remodeling was closely associated with improved metabolic phenotypes in UMP-treated mice, the current study does not establish a causal relationship. Future studies using fecal microbiota transplantation, antibiotic-treated mice, germ-free mouse models, or targeted functional prediction and validation analyses are required to determine whether gut microbiota changes are necessary or sufficient for the metabolic benefits of UMP.

At the metabolic level, elevated fecal FFAs indicate reduced intestinal fatty acid absorption, which directly lowers lipid influx into the liver and adipose tissue, consistent with the observed decreases in hepatic and serum lipids (Section 3.4 and Section 3.5) [58]. Mechanistically, this effect could be attributed to UMP’s action in downregulating key intestinal fatty acid transporters (CD36, FATP4) or to microbiota-dependent remodeling of the intestinal lipid microenvironment that collectively inhibits fatty acid uptake [48,58,59]. Furthermore, UMP uniquely reprogrammed bile acid metabolism, evidenced by normalization of total bile acid excretion and enrichment of secondary bile acids such as 7-ketolithocholic acid. Regulation of bile acid signaling through FXR and TGR5 pathways has been recognized as a crucial interface between gut microbiota and host metabolic homeostasis [60,61]. UMP treatment elevated levels of serum arachidonic acid (AA), a major n-6 fatty acid and linoleic acid derivative. Enhanced AA metabolism within the fatty acid oxidation pathway optimizes lipid turnover. This acceleration aligns with the decreased serum and hepatic TG/TC levels (Section 3.4 and Section 3.5), indicating that an accelerated AA turnover boosts overall lipid catabolism and thereby contributes to the systemic hypolipidaemic effect [62,63]. Conversely, the reduction in serum D-glucose corresponds directly to the improved glucose tolerance and insulin sensitivity (Section 3.3), confirming enhanced systemic glucose homeostasis. The enrichment of pathways, particularly ABC transporters and glucagon signaling, highlights a targeted hepatic-centric mechanism for lipid regulation. ABC transporters are essential for cholesterol homeostasis through their mediation of cellular lipid efflux [64,65]. Enhanced glucagon-receptor signalling was closely associated with improved lipid metabolism. The liver is the central site of glucagon action. By orchestrating hepatic lipid metabolism, glucagon may attenuate hepatic lipid accumulation and the subsequent secretion of lipids [66]. The KEGG-enriched biosynthesis of cofactors pathway may reflect broader improvement in cellular redox reactions, mitochondrial substrate oxidation, and amino acid-related energy metabolism [67,68]. These changes are consistent with the observed improvement in insulin sensitivity and reduced hepatic lipid accumulation. In parallel, ABC transporters are key regulators of cholesterol efflux, bile acid transport, lipid trafficking, and metabolic detoxification [69]. Therefore, the modulation of ABC transporter pathways may be mechanistically linked to reduced serum TC and LDL-C levels, decreased hepatic lipid deposition, and improved bile acid homeostasis in UMP-treated mice. These pathway-level changes indicate that UMP exerts systemic metabolic benefits through coordinated regulation of lipid transport, bile acid signaling, and energy metabolism rather than through a single metabolite.

Correlation analysis linking microbial taxa with metabolites further demonstrates the lipid-lowering effect of UMP. *Ileibacterium* exhibited a strong positive correlation with quercetin-3-O-sophoroside, underscoring its involvement in glucose and lipid metabolism. Quercetin-3-O-sophoroside exerts its effects by modulating the activity of glucose-metabolizing enzymes, inhibiting lipogenesis and lipid accumulation, and regulating the gut microbiota [70]. Both *Ileibacterium* and *norank_f_Muribaculaceae* exhibited significant positive correlations with 2-hydroxybutyric acid, a metabolite that functions in fatty acid metabolism and possesses the capacity to improve insulin sensitivity and suppress inflammation [71]. In contrast, *Ileibacterium* and *norank_f_Muribaculaceae* exhibited negative correlations with pantothenic acid, which is the metabolic precursor of coenzyme A (CoA) and participates mainly in the form of CoA in the metabolism of carbohydrates, lipids and proteins [72]. The decrease in serum pantothenic acid may reflect an elevated utilization rate by the microbiota, driving its conversion into CoA to support energy metabolism. These correlations establish a microbiota-metabolite-host regulatory axis: UMP enriches beneficial genera such as *Ileibacterium* and *norank_f_Muribaculaceae* that produce SCFAs and modify fatty-acid and protein metabolism, leading to changes in serum metabolites that improve glucose metabolism, reduce lipid accumulation, and alleviate inflammation. This axis is the core mechanism underlying UMP’s anti-obesity effects. Moreover, the positive association between Faecalibacterium and L-ascorbic acid 2-sulfate may indicate a potential interaction between microbial anti-inflammatory capacity and host antioxidant metabolism. Faecalibacterium is commonly associated with intestinal health and SCFA production [73,74], whereas L-ascorbic acid 2-sulfate represents a sulfated derivative related to antioxidant homeostasis [75,76]. Therefore, their correlation may reflect a coordinated microbiota-metabolite response that helps counteract HFD-induced oxidative and inflammatory stress. Nevertheless, this association should be interpreted cautiously because correlation analysis does not establish causality.

Collectively, these results indicate that UMP exerts its anti-obesity effects through multi-targets, synergistic mechanism involving gut microbiota modulation, metabolite regulation, improved barrier function, and systemic metabolic reprogramming. UMP orchestrates a microbiota-metabolite-host axis that underpins its comprehensive anti-obesity phenotype, highlighting its potential as a novel dietary protein for preventing and managing obesity-related metabolic disorders. These findings extend previous work showing dietary interventions can modulate metabolic outcomes via gut microbiota-regulated pathways [77]. Despite these promising findings, several limitations warrant consideration. The strong correlations we observe between specific bacterial taxa such as *Ileibacterium*, key serum metabolites including 2-hydroxybutyric acid, and host metabolic phenotypes suggest but do not prove causality. Fecal microbiota transplantation (FMT) from UMP-treated mice to naive HFD-fed recipients would provide direct evidence for the causal role of the microbiota in mediating UMP’s metabolic benefits.

## 5. Conclusions

In summary, this study demonstrated that ultrafiltered mulberry leaf albumin-type protein (UMP) effectively alleviated HFD-induced obesity and metabolic disorders in mice. UMP supplementation reduced body weight gain, improved insulin sensitivity, ameliorated dyslipidemia, alleviated hepatic steatosis, protected colonic morphology, and decreased circulating LPS levels. Moreover, UMP restored gut microbial diversity, enriched beneficial bacterial taxa, increased SCFA production, and partially normalized bile acid metabolism. Untargeted metabolomics further revealed that UMP reshaped multiple metabolic pathways associated with lipid metabolism, glucose utilization, and metabolic homeostasis. Correlation analysis suggested that these beneficial effects may be mediated through a microbiota-metabolite-host interaction network. Collectively, these findings indicate that UMP has strong potential as a functional dietary protein for the prevention and management of obesity-related metabolic disorders.

## Figures and Tables

**Table 1:** Nutritional composition of the UMP.

**Composition**	**Concentration (g/100 g)**
Protein content	87.12 ± 0.52
Polyphenols (gallic acid equivalent)	2.52 ± 0.00
Polysaccharides	8.21 ± 1.49

**Table 2:** Percentage of secondary structure of the UMP (%).

***β*** **-Sheet**	**Random Coil**	***α*** **-Helix**	***β*** **-Turn**
12.64 ± 1.68	19.89 ± 2.07	25.21 ± 1.70	42.26 ± 2.64

**Table 3:** UMP-mediated alterations in serum biochemical parameters in mice.

**Parameters**	**ND**	**HFD**	**LUMP**	**HUMP**
TG (mmol/L)	0.79 ± 0.09 ^b^	1.03 ± 0.27 ^a^	0.78 ± 0.12 ^b^	0.80 ± 0.08 ^b^
TC (mmol/L)	3.20 ± 0.74 ^c^	5.71 ± 0.41 ^a^	4.73 ± 0.91 ^b^	3.89 ± 1.13 ^bc^
HDL-C (mmol/L)	2.52 ± 0.23 ^a^	2.70 ± 0.11 ^a^	2.63 ± 0.25 ^a^	2.70 ± 0.24 ^a^
LDL-C (mmol/L)	0.38 ± 0.04 ^b^	0.67 ± 0.09 ^a^	0.45 ± 0.13 ^b^	0.36 ± 0.08 ^b^
ALT (U/L)	34.22 ± 3.79 ^c^	111.72 ± 25.44 ^a^	72.58 ± 21.80 ^b^	69.45 ± 19.30 ^b^
AST (U/L)	151.37 ± 27.69 ^b^	225.08 ± 74.40 ^a^	169.39 ± 38.47 ^ab^	171.69 ± 48.35 ^ab^
Cre (µmol/L)	36.32 ± 3.03 ^c^	50.83 ± 3.75 ^a^	46.39 ± 3.95 ^b^	45.57 ± 1.91 ^b^
BUN (mmol/L)	12.42 ± 1.76 ^a^	7.40 ± 0.62 ^c^	7.52 ± 0.80 ^c^	9.57 ± 0.93 ^b^
LPS (ng/mL)	2.08 ± 0.12 ^b^	4.48 ± 1.46 ^a^	2.74 ± 0.58 ^b^	2.48 ± 0.51 ^b^

Values are presented as mean ± SD (*n* = 7). Within the same row, different superscript letters (a–c) denote statistically significant differences among groups (*p* < 0.05).

## Data Availability

The original contributions presented in this study are included in the article/Supplementary Materials. Further inquiries can be directed to the corresponding authors.

## Supplementary Materials

The following supporting information can be downloaded at: https://www.mdpi.com/article/10.3390/foods15162774/s1, Figure S1 The SDS-PAGE of mulberry leaves protein, the original image of Figure 1A. Figure S2 The Effects of the UMP on liver injury in mice, the original image of Figure 4B,C. Figure S3 The Effects of UMP on colonic morphology and SCFAs levels in mice, the original image of Figure 5A.
