# Supplementary material for: Ultrafiltered Mulberry (Morus alba L.) Leaf Albumin-Type Protein Attenuates High-Fat Diet-Induced Obesity in Mice by Remodeling Gut Microbiota and Metabolic Homeostasis
Source: Foods. 2026 Aug 7;15(16):2774. doi: 10.3390/foods15162774 (PMC13511888; doi:10.3390/foods15162774)
Supplement: Supplementary file 1 [file foods-15-02774-s001.zip › foods-4386392-supplementary.pdf]

Supplementary Information for

Ultrafiltered Mulberry (*Morus alba L.*) Leaf Albumin Attenuates High-Fat Diet-Induced Obesity in Mice by Remodeling Gut Microbiota and Metabolic Homeostasis

Leyi Yu[a, b1], Kaiwen Luo[b1], Dongjun He[b1], Guoxing Yu[b], Yu Yang[b], Hong Yao[b], Chongzhen Sun[c*], Xiyang Wu[b*]

Affiliations

[a] School of Life Sciences, Zhejiang Chinese Medical University, Hangzhou 310053, Zhejiang, PR China;

[b] Department of Food Science and Engineering, Jinan University, Guangzhou 510632, PR China;

[c] School of Public Health, Guangdong Pharmaceutical University, Jianghai Avenue 283, Haizhu District, Guangzhou 510006, PR China.

Leyi Yu: 20241156@zcmu.edu.cn; Kaiwen Luo: 15728054368@163.com; Dongjun He: 13202457231@163.com; Guoxing Yu: cusiyu@163.com; Yu Yang: 13427519502@163.com; Hong Yao: whyhsss@outlook.com

*Corresponding author: Chongzhen Sun: sunchongzhen@gdpu.edu.cn; Xiyang Wu: tkentwu@jnu.edu.cn

[1] The authors contribute equally to this paper.

Supplementary materials include the following:

Total pages: 9; total figures: 3; total tables: 0;

Figure S1 The SDS-PAGE of mulberry leaves protein, the original image of Figure 1A.

Figure S2 The Effects of the UMP on liver injury in mice, the original image of Figure 4B,C.

Figure S3 The Effects of UMP on colonic morphology and SCFAs levels in mice, the original image of Figure 5A.

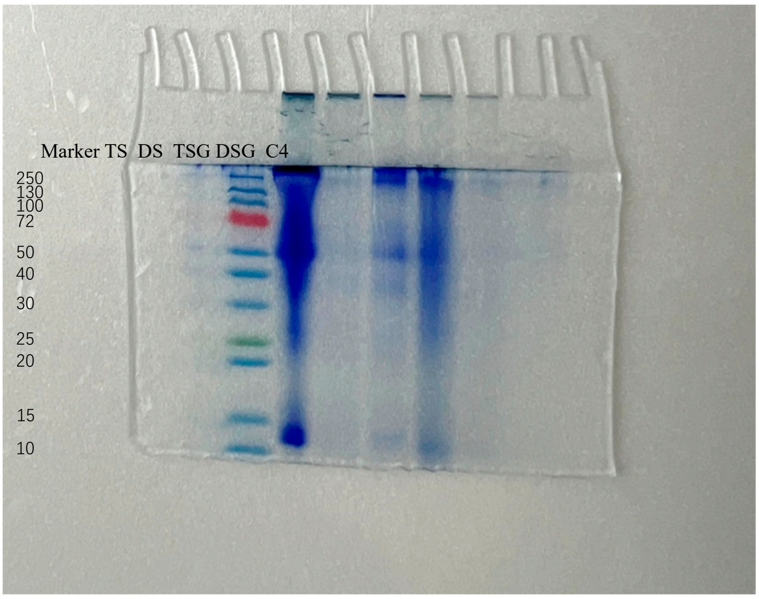

Figure S1 The SDS-PAGE of mulberry leaves protein, the original image of Figure 1A. Protein samples were separated on a 12% gel and stained with Coomassie Blue. Lanes: TS, DS, C4 (albumin fractions from three mulberry varieties); TSG (globulin fraction from TS); DSG (globulin fraction from DS). Molecular weight markers (kDa) are indicated on the left.

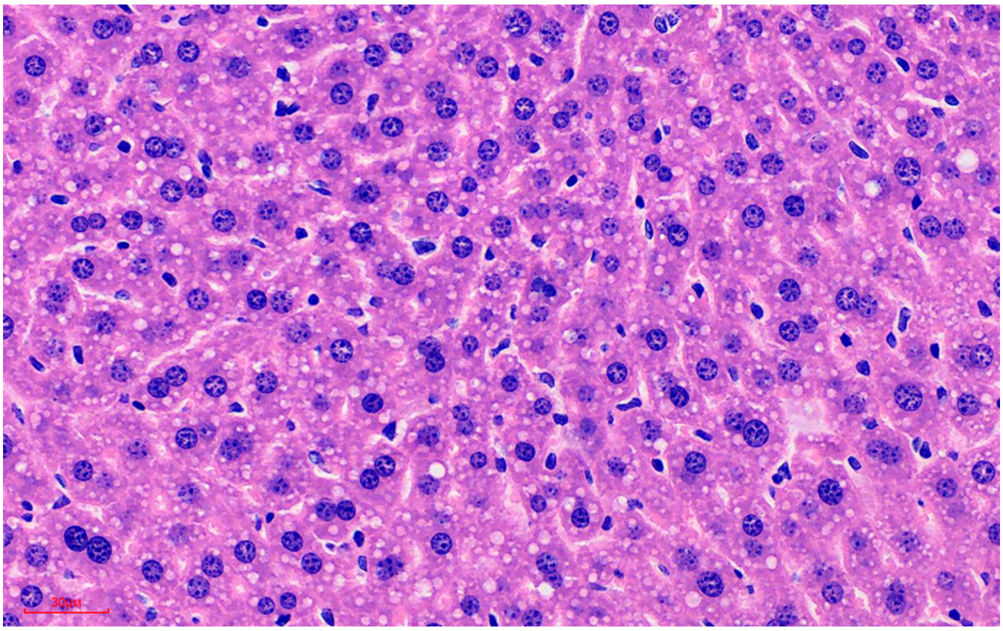

Figure 4 B_1

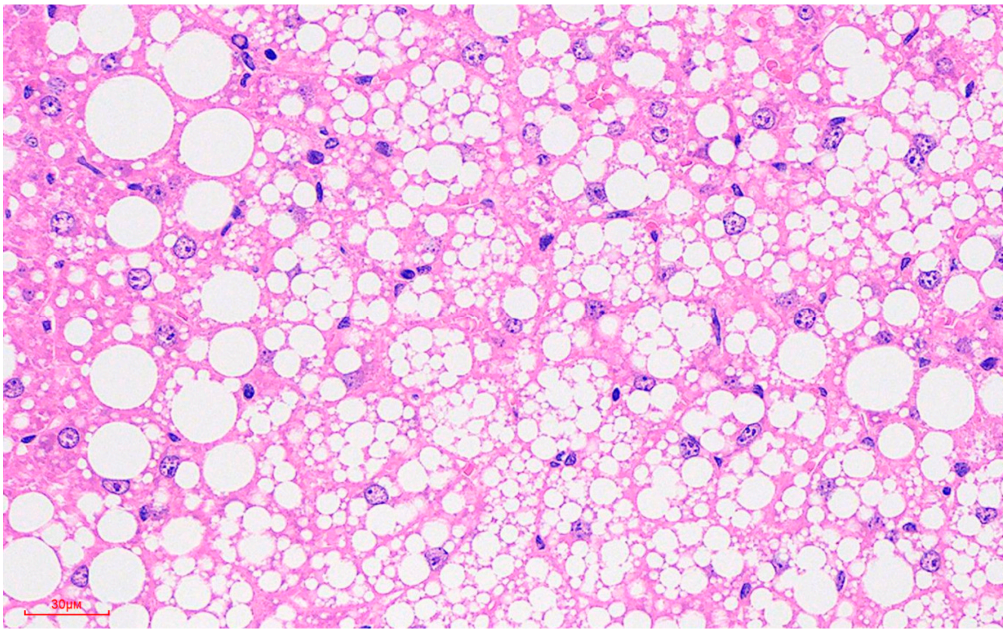

Figure 4 B_2

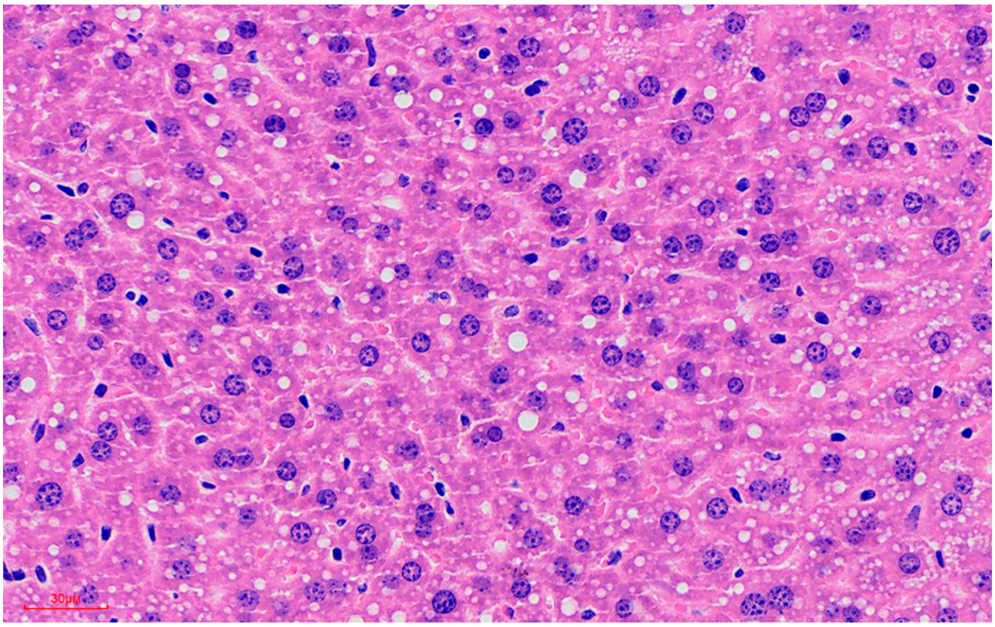

Figure 4 B_3

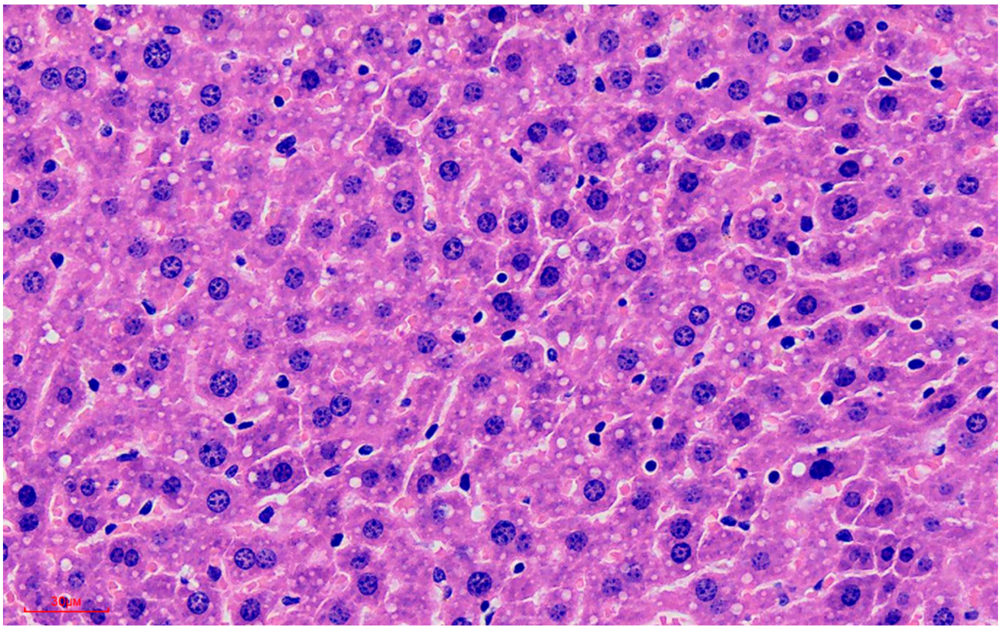

Figure 4 B_4

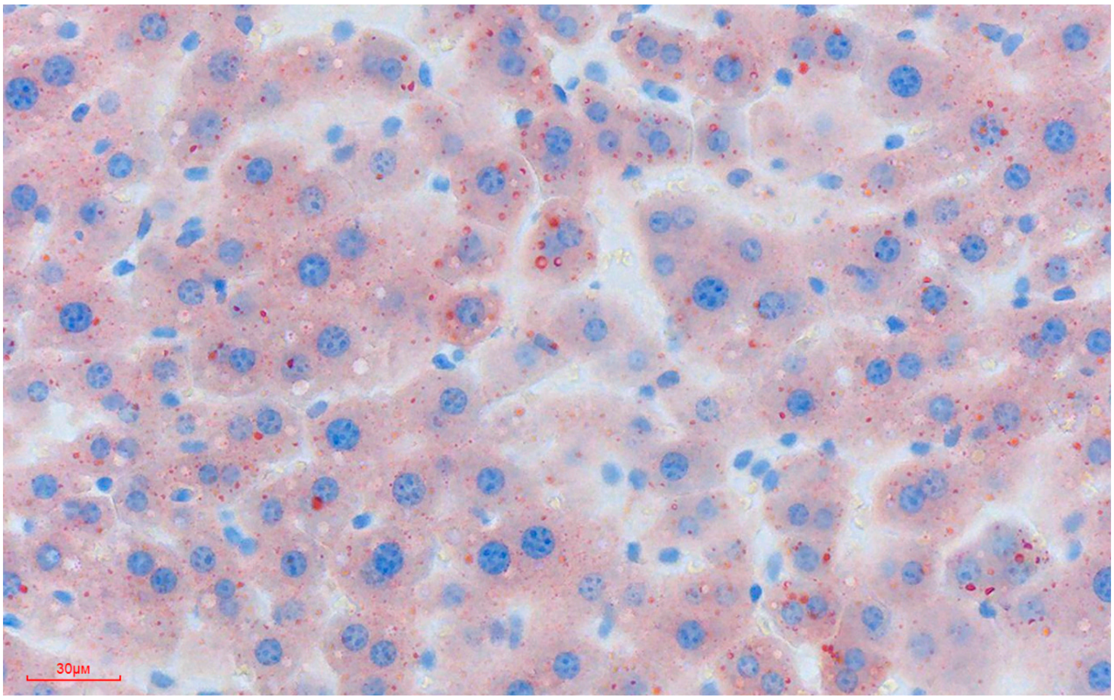

Figure 4 C_1

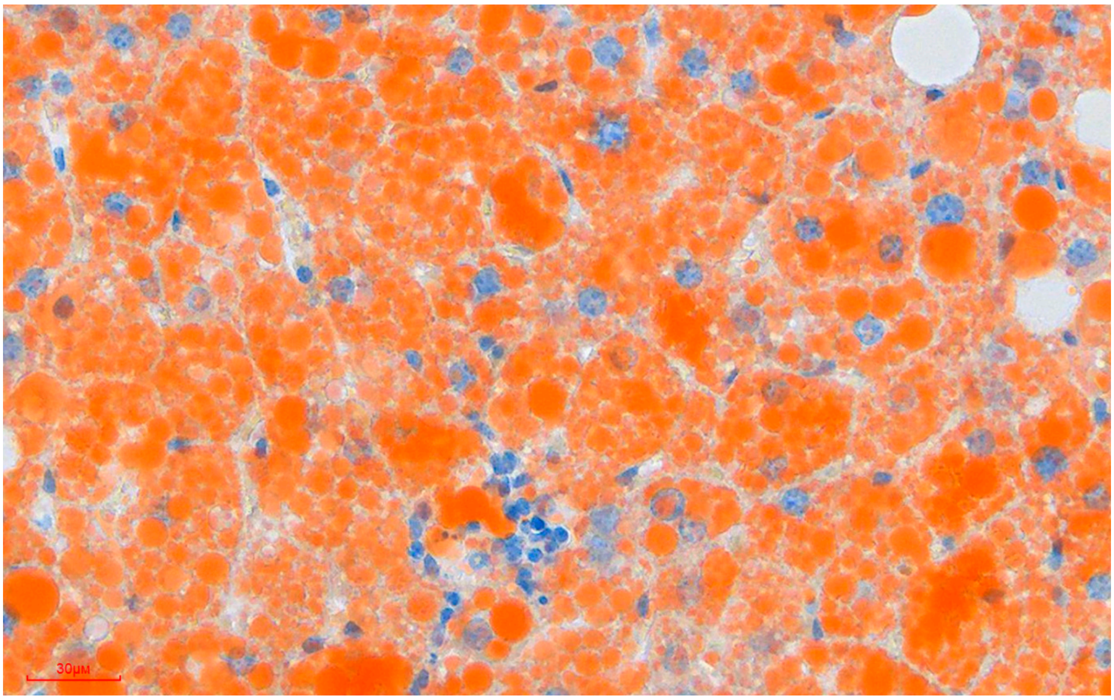

Figure 4 C_2

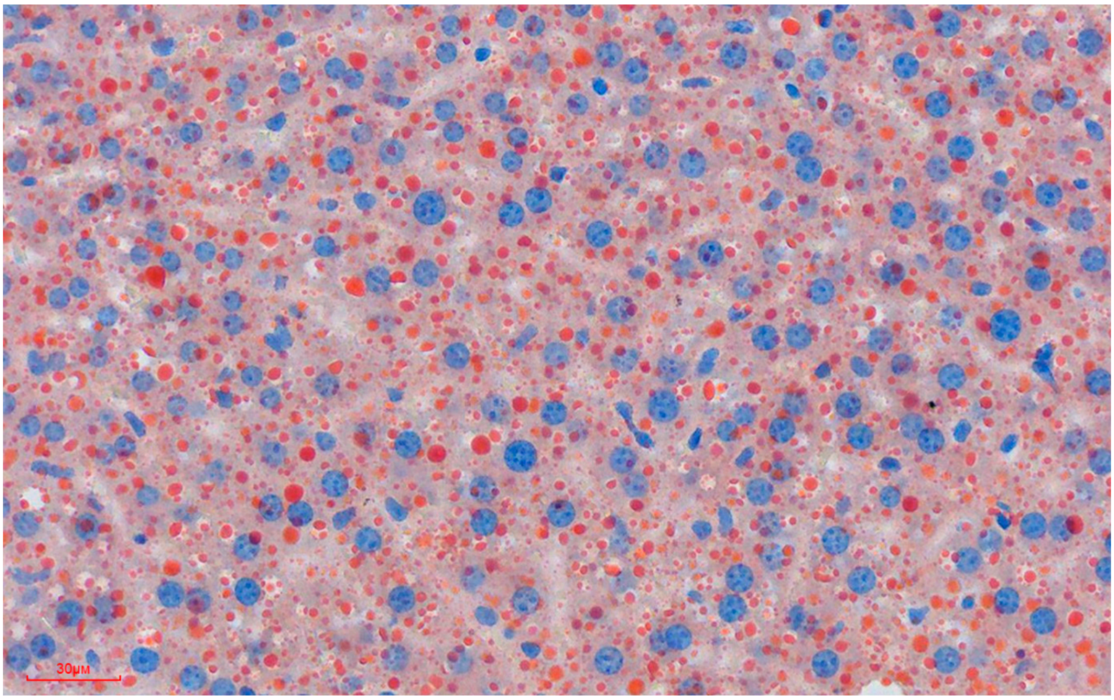

Figure 4 C_3

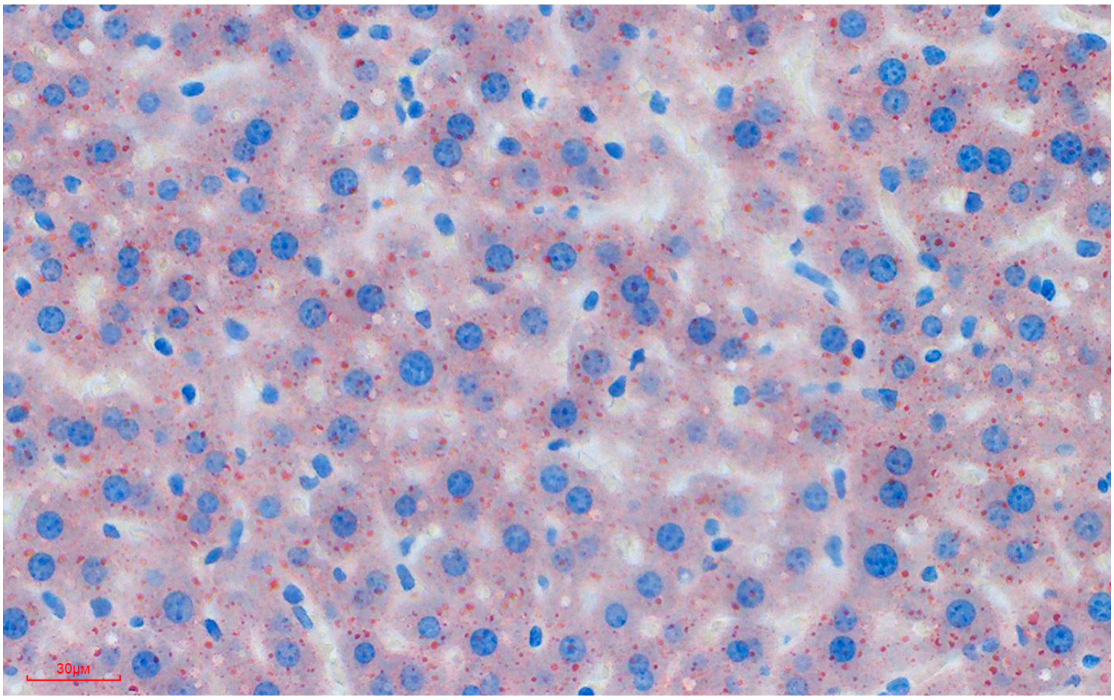

Figure 4 C_4

Figure S2 The Effects of the UMP on liver injury in mice, the original image of Figure 4B,C. (B) H&E staining (40×); (C) Oil Red O staining (40×).

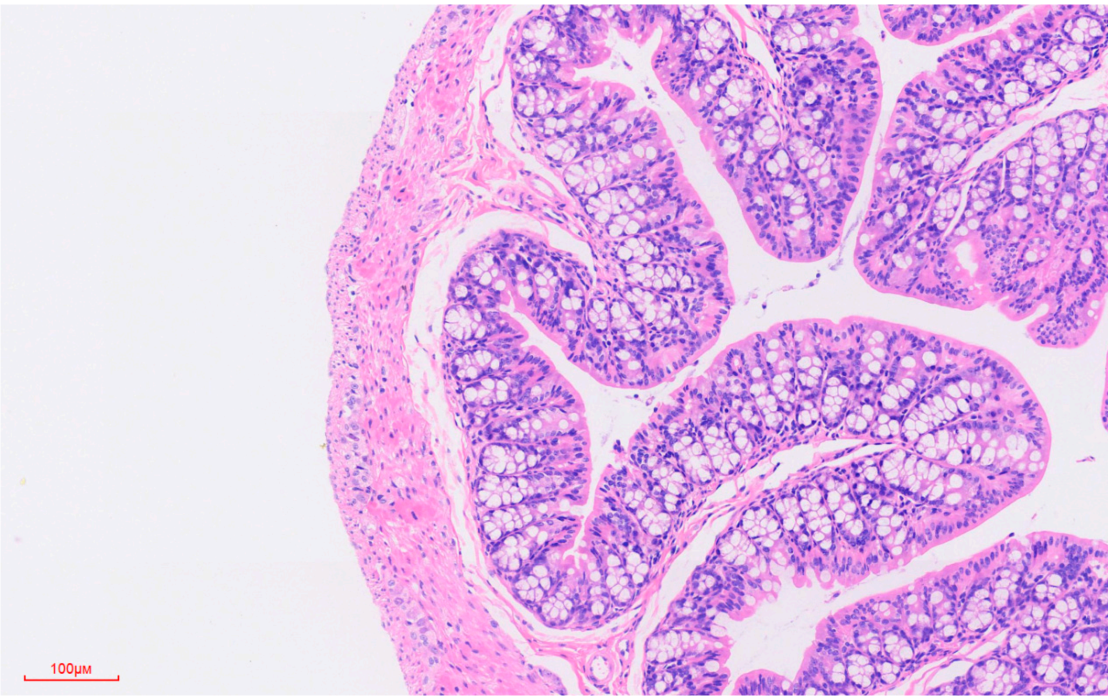

Figure 5 A_1

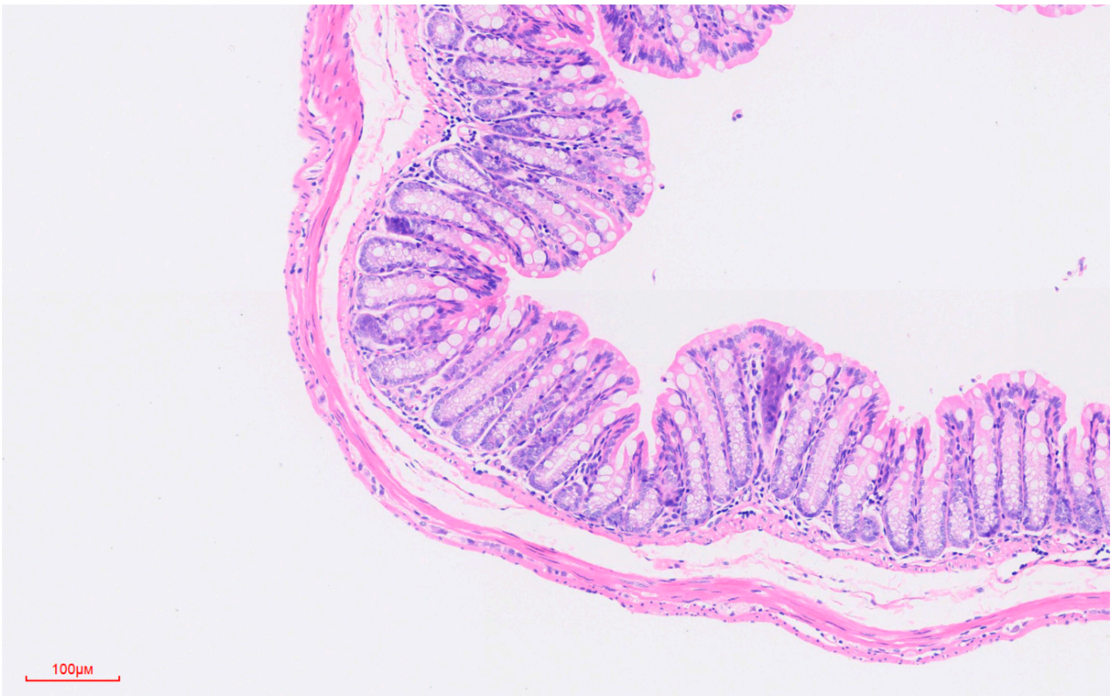

Figure 5 A_2

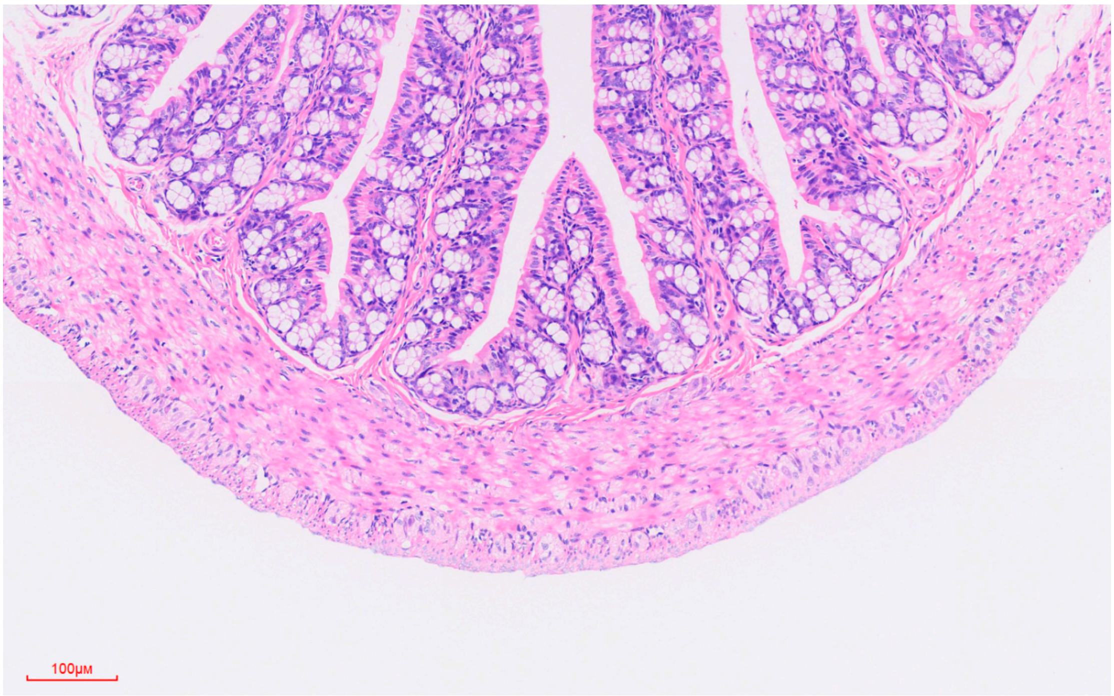

Figure 5 A_3

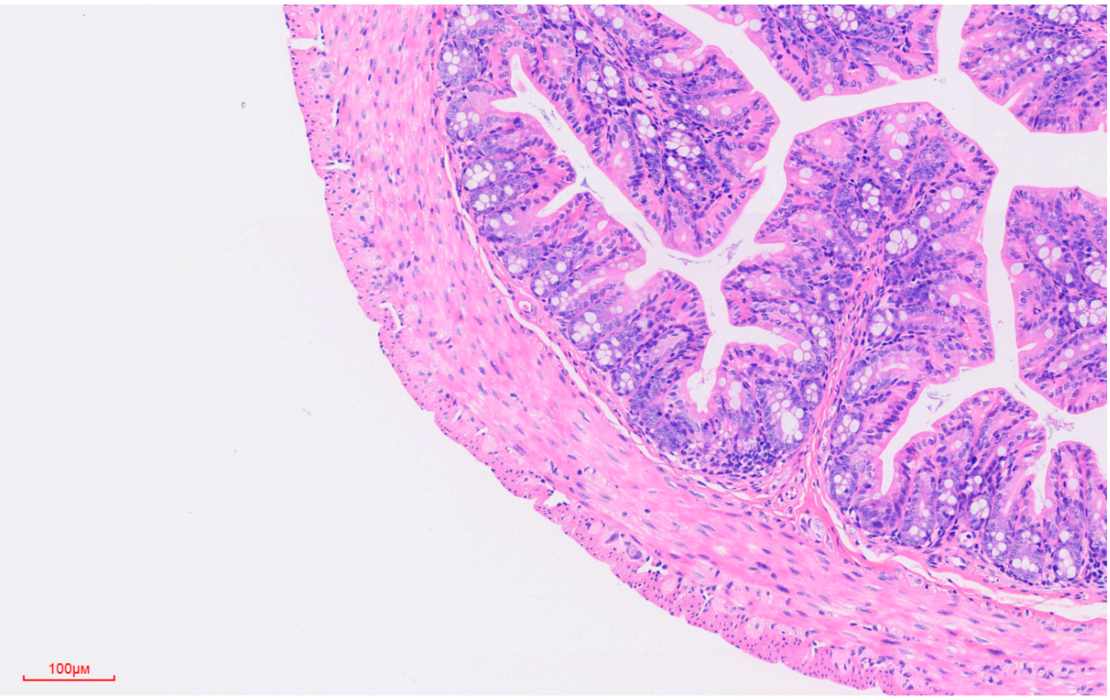

Figure 5 A_4

Figure S3 The Effects of UMP on colonic morphology and SCFAs levels in mice, the original image of Figure 5A. (A) H&E staining (12×).
